# Glycerol and reuterin-producing *Limosilactobacillus reuteri* enhance butyrate production and inhibit *Enterobacteriaceae* in broiler chicken cecal microbiota PolyFermS model

**DOI:** 10.1186/s12866-023-03091-6

**Published:** 2023-12-05

**Authors:** Paul Tetteh Asare, Anna Greppi, Annelies Geirnaert, Alessia Pennacchia, Angela Babst, Christophe Lacroix

**Affiliations:** 1 Department of Health Sciences and Technology, Laboratory of Food Biotechnology, Institute of Food, Nutrition and Health, ETH Zürich, LFV D 20, Schmelzbergstrasse 7, CH-8042 Zurich, Switzerland; 2 Present address: Gnubiotics Sciences SA, Epalinges, Switzerland

**Keywords:** Glycerol, Chicken cecal microbiota, In vitro model, *L. reuteri*, Butyrate, Reuterin

## Abstract

**Background:**

Administering probiotic strains of *Limosilactobacillus reuteri* to poultry has been shown to improve poultry performance and health. Some strains of *L. reuteri* taxa can produce reuterin, a broad-spectrum antimicrobial compound from glycerol conversion, with high inhibitory activity against enterobacteria. However, little is known about the metabolism of glycerol in the complex chicken cecal microbiota nor the effect of glycerol, either alone or combined with *L. reuteri* on the microbiota. In this study, we investigated the effect of *L. reuteri* PTA5_F13, a high-reuterin-producing chicken strain and glycerol, alone or combined, on broiler chicken cecal microbiota composition and activity using the continuous PolyFermS model recently developed to mimic chicken cecal fermentation.

**Methods:**

Three independent PolyFermS chicken cecal microbiota models were inoculated with immobilized cecal microbiota from different animals and operated continuously. The effects of two additional levels of glycerol (50 and 100 mM) with or without daily supplementation of chicken-derived *L. reuteri* PTA5_F13 (10^7^ CFU/mL final concentration) were tested in parallel second-stage reactors continuously inoculated with the same microbiota. We analyzed the complex chicken gut microbiota structure and dynamics upon treatment using 16S rRNA metabarcoding and qPCR. Microbiota metabolites, short-chain and branched-chain fatty acids, and glycerol and reuterin products were analyzed by HPLC in effluent samples from stabilized reactors.

**Results:**

Supplementation with 100 mM glycerol alone and combined with *L. reuteri* PTA5_F13 resulted in a reproducible increase in butyrate production in the three modelled microbiota (increases of 18 to 25%). Glycerol alone resulted also in a reduction of *Enterobacteriaceae* in two of the three microbiota, but no effect was detected for *L. reuteri* alone. When both treatments were combined, all microbiota quantitatively inhibited *Enterobacteriaceae*, including in the last model that had very high initial concentrations of *Enterobacteriaceae*. Furthermore, a significant 1,3-PDO accumulation was measured in the effluent of the combined treatment, confirming the conversion of glycerol via the reuterin pathway. Glycerol supplementation, independent of *L. reuteri* addition, did not affect the microbial community diversity.

**Conclusions:**

Glycerol induced a stable and reproducible butyrogenic activity for all tested microbiota and induced an inhibitory effect against *Enterobacteriaceae* that was strengthened when reuterin-producing *L. reuteri* was spiked daily. Our in vitro study suggests that co-application of *L. reuteri* PTA5_F13 and glycerol could be a useful approach to promote chicken gut health by enhancing metabolism and protection against *Enterobacteriaceae*.

**Supplementary Information:**

The online version contains supplementary material available at 10.1186/s12866-023-03091-6.

## Background

The chicken cecum is a densely populated compartment of the gastrointestinal tract (GIT) that harbors a complex microbial community dominated by the bacterial phyla Firmicutes, Bacteroidetes and Proteobacteria [1, 2]. Cecal microbiota supports chicken nutrition by metabolizing undigestible dietary compounds, producing metabolites such as short-chain fatty acids (SCFA) and vitamins, and also protecting against infection by producing a variety of antimicrobial compounds [3–5]. However, the chicken GIT still frequently harbors pathogens such as *Campylobacter jejuni* and *Salmonella enterica* that can be transmitted to humans by handling and consuming improperly cooked meat [6]. Hence, there is growing interest in developing feed supplements that can enhance the protective function of the chicken gut microbiota and prevent invasion and colonization by enteropathogens [4]. Thus, natural growth promoters, such as probiotics, prebiotics and phytobiotics, are promising alternatives to antibiotics in poultry production [7, 8].

*Limosilactobacillus reuteri* (formerly *Lactobacillus reuteri*) is the most abundant *Lactobacillaceae* species in the chicken crop and cecum [9, 10]. *L. reuteri* lacks extracellular polysaccharide-degrading enzymes which reflects its adaptation to nutrient-rich segments of the upper intestine of animals [11–13]. Previous works in broiler chickens showed that supplementing *L. reuteri* in feed or by oral gavage decreased the relative abundance of the *Proteobacteria* and *Enterobacteriaceae* families [14, 15]*.* Different mechanisms have been suggested for the probiotic effect of *L. reuteri*, including stimulating the immune system, competitive exclusion, and producing antimicrobial compounds such as organic acids and reuterin [15–17].

**Fig. 1:**
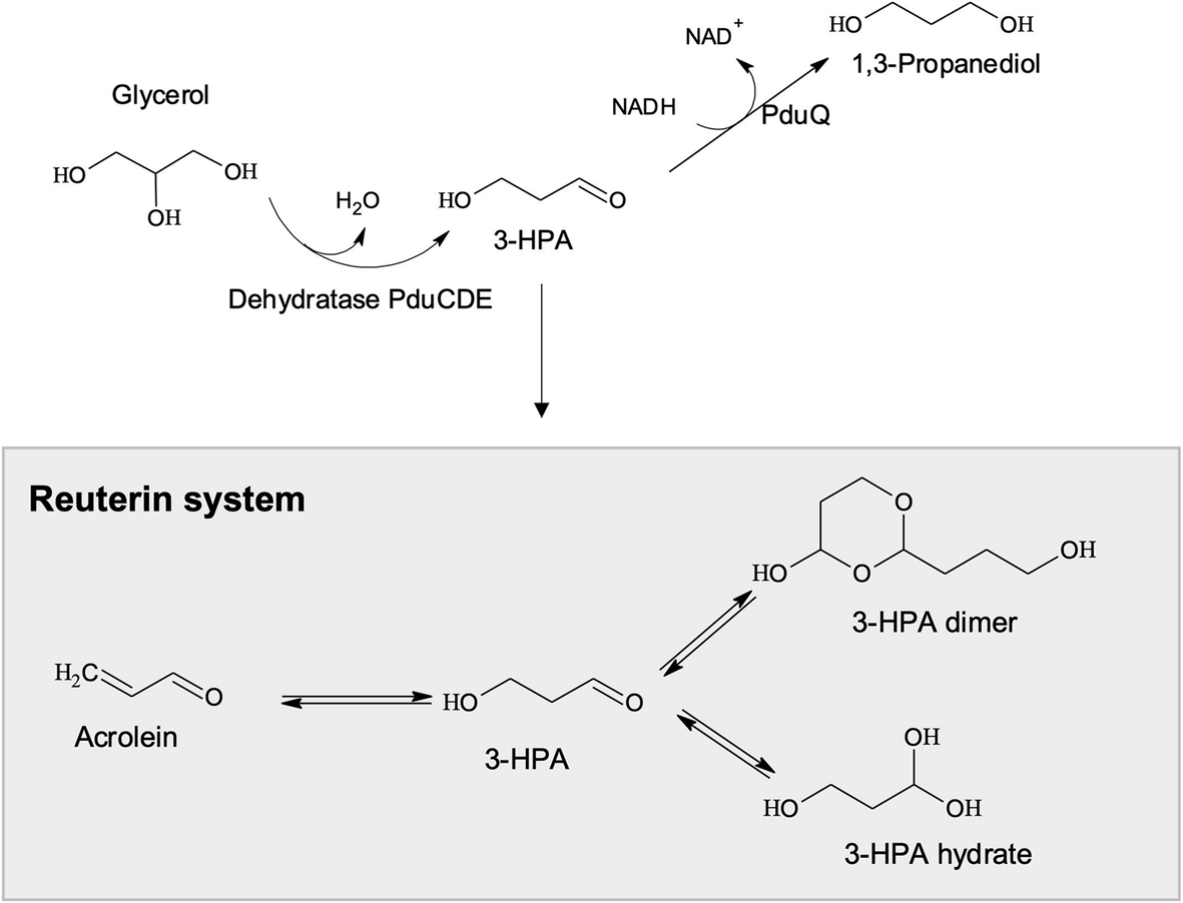
Glycerol metabolism by reuterin-producing* L. reuteri*. Anaerobic metabolism of glycerol by reuterin-producing* L. reuteri *to 3-hydroxypropionaldehyde (3-HPA) and further to 1,3-propanediol (1,3-PDO). In an aqueous environment, 3-HPA is quickly dimerised and hydrated to form HPA-dimer and HPA-hydrate and also spontaneously dehydrates to acrolein together form the reuterin system. PduQ, 1,3-PDO dehydrogenase

Reuterin is a multi-compound system produced from glycerol conversion and consisting of 3-hydroxypropionaldehyde (3-HPA), 3-HPA hydrate, 3-HPA dimer and acrolein (Fig. 1). Reuterin is a potent broad-spectrum antimicrobial compound active over a wide pH range with antimicrobial activity against intestinal bacteria; in particular, reuterin is effective at low concentrations against members of the *Enterobacteriaceae* family, including *Campylobacter*, *Salmonella* and *Escherichia coli* [18–20]. Strains of the gut commensals, including *L. reuteri*, *Anaerobutyricum hallii*, *Flavonifractor plautii*, and *Blautia obeum*, along with members of less favorable genera such as *Klebsiella*, *Enterobacter, Citrobacter* and *Salmonella*, harbor glycerol/diol dehydratases that catalyze glycerol conversion to 3-HPA [21, 22]. Chicken isolates of *L. reuteri* must possess the glycerol/diol dehydratase PduCDE (EC 4.2.1.30) operon in their genome to convert glycerol to reuterin [23, 24]. However, for most producer strains, 3-HPA is immediately reduced to 1,3-propanediol (1,3-PDO), but certain strains of *L. reuteri* can excrete 3-HPA in a low-glucose environment, as is found in the gut [25]. Further, there is no data on the synthesis and effects of reuterin in the complex chicken cecal microbiota. Glycerol is frequently added to animal feed as an energy source [26] and to improve pellet quality [27]. Due to its sweet taste and small molecular size, crude glycerol in the diet has been reported to increase feed intake and improve body weight gain and feed conversion efficiency of broiler chickens [28, 29]. However, little is known about glycerol metabolism in the chicken gut microbiota and its possible modulatory effect on microbiota composition and metabolism.

In this study, we investigated the effect of glycerol on modelled chicken cecal microbiota, independent of host factors, by using the recently developed and validated continuous chicken cecal PolyFermS model [30]. We evaluated colonization of the chicken-derived strain of *L. reuteri* PTA5_F13, selected for its high reuterin production, and the effects of strain and glycerol, either alone or combined, on microbiota composition and activity using 16S rRNA metabarcoding and quantitative PCR and HPLC analysis of effluent samples, respectively.

## Results

### PolyFermS fermentations 

Three continuous PolyFermS fermentations inoculated with different immobilized chicken cecal microbiota were carried out to test the effects of glycerol and reuterin-producing *L. reuteri* PTA5_F13 and the combination thereof. Description baseline data of the three models (F1A, F2 and F3) that were used to develop and validate the chicken cecal PolyFermS model were previously presented in detail [30]. The IRs of the three models were respectively operated for total times of 13, 70 and 82 days for F1, F2 and F3. The treatment test periods corresponded to days 8–13, 35–42 and 38–46 of the operation of the respective IRs. Briefly, the cecal microbiota of the three donors for F1, F2 and F3, respectively, were all dominated by the bacterial phylum Firmicutes (89.4%, 95.5 vs 98.2%), followed by Bacteroidetes (4.6%, 3.5% vs 0.2%), Proteobacteria (5.3%, 0.26% vs 2.3%), and Actinobacteria (0.13%, 0.25% vs 0.70%). After initial stabilization, IRs inoculated with cecal beads had stable metabolite and microbial composition in their effluents over the entire operation time. Moreover, reproducible metabolic profiles were observed in all second-stage reactors of each model and were representative of the donor chicken cecum [30]. However, some variations in microbiota composition were observed among the second-stage reactors TRs and CR during stabilization periods of F2 and F3, tentatively explained by the limited accuracy of pumps feeding all second-stage reactors with a constant 5% inoculum rate of effluent from IR (corresponding to a very low inoculum flow rate of 8.3 mL/h), which is heterogeneous and contains particulates.

### Impact of glycerol on chicken cecal microbiota metabolites 

Metabolites (SCFAs, BCFAs, intermediate products and 1,3-PDO) were analyzed in the fermentation effluents using HPLC. During F1, a high reproducibility of microbiota composition and activity in all CR and TRs during pretreatment were observed (Fig. S1). Therefore, TRs could directly be compared to CR during the treatment period for F1 (Table 1). As expected, adding glycerol (50G and 100G) to the TRs significantly increased total metabolite concentrations by + 11.7 mM (*p* < 0.05), and + 49.3 mM (*p* < 0.001), respectively, compared to CR. Both treatments increased butyrate (*p* < 0.05), from 26.7 mM in CR to 38.5 mM and 46.7 mM for 50G and 100G, respectively. We also measured a significant dose-dependent decrease of acetate (-12.2 and -16.7 mM for 50G and 100G, respectively) and propionate (- 4.5 and - 6.4 mM) and accumulation of 1,3-PDO, only when glycerol was added (Table 1 and Fig. S1) compared to CR. Lactate was not detected and glycerol had no effect on the intermediate products succinate and valerate.

**Table 1:** Effect of glycerol supplementation on the metabolic activity of chicken cecal microbiota during in vitro F1

Reactors	Concentration (mM) ± SD							
	**Acetate**	**Butyrate**	**Propionate**	**Succinate**	**Valerate**	**1,3 – PDO**	**Total BCFAs**	**Total metabolites**
**CR**								
Treatment	75.2 ± 0.9	26.7 ± 2.6	17.9 ± 0.3	2.7 ± 0.6	3.5 ± 1.3	0.0 ± 0.0	13.4 ± 0.1	139.4 ± 4.4
**50G**								
Treatment	63.0 ± 4.8	38.5 ± 5.2	13.4 ± 1.3	1.7 ± 0.2	4.3 ± 0.9	19.0 ± 0.4	10.8 ± 0.2	151.1 ± 2.9
Delta	**- 12.2**	** + 11.7**	**- 4.5**	- 0.9	+ 0.8	** + 19.0**	**- 2.5**	** + 11.7**
*P* value with CR	**0.0128**	**0.0240**	**0.0049**	0.0745	0.4453	-	**0.0001**	**0.0187**
**100G**								
Treatment	58.5 ± 0.8	46.7 ± 6.0	11.5 ± 1.3	5.4 ± 2.3	5.2 ± 1.0	49.4 ± 0.7	11.8 ± 0.3	188.7 ± 7.4
Delta	**- 16.7**	** + 19.9**	**- 6.4**	+ 2.7	** + 1.7**	** + 49.4**	**- 1.5**	** + 49.3**
*P* value with CR	**0.000018**	**0.0062**	**0.0013**	0.1261	**0.1529**	**-**	**0.0030**	**0.0005**

Data are means ± SD for the last 3 days of each treatment period. Delta corresponds the difference between treatment and control reactor (CR). *P* value is calculated for the pairwise comparison of the last 3 days of treatment and CR by unpaired *t*-test. CR, Control reactor; 50G, 50 mM glycerol; 100G, 100 mM glycerol

The high dose of glycerol (100 mM) was repeated in F2 and F3, and data from these treatments were compared to data from pre-treatment stabilization of the same reactor (Table 2). Upon addition of glycerol (100G), a reproducible significant increase (*p* < 0.05) in total SCFA (+ 9.4 mM and + 22.3 mM) and butyrate (+ 23.5 mM and + 38.0 mM), and a significant decrease in acetate (- 34.8 mM and - 32.8 mM) was measured compared to the respective pre-treatment for F2 and F3, respectively. Propionate, succinate, and valerate remained unchanged during glycerol supplementation, while PDO was only produced when glycerol was supplemented (+ 23 mM and + 22.3 mM for F2 and F3, respectively) (Tables 2 and 3 and Figs. S2 and S3).

**Table 2:** Effect of glycerol supplementation and *L. reuteri* PTA5_F13 on the metabolic activity of chicken cecal microbiota during in vitro F2

Reactors	Mean metabolite concentration (mM) ± SD								Ratios of Total SCFAs (%)		
	**Acetate**	**Butyrate**	**Propionate**	**Succinate**	**Valerate**	**1,3 – PDO**	**Total BCFAs**	**Total metabolites**	**Acetate**	**Butyrate**	**Propionate**
**CR**											
Pre-treatment	91.2 ± 0.9	28.8 ± 0.1	23.1 ± 1.0	7.1 ± 0.8	8.3 ± 0.1	0.0 ± 0.0	14.8 ± 0.2	174.1 ± 1.9	63.7 ± 0.7	20.1 ± 0.1	16.2 ± 0.7
Treatment	95.9 ± 1.0	27.2 ± 0.6	21.9 ± 0.3	6.8 ± 0.2	7.1 ± 0.1	0.0 ± 0.0	14.9 ± 0.2	174.0 ± 1.3	66.1 ± 0.2	15.1 ± 0.2	18.8 ± 0.4
Treatment – Pre treatment	** + 4.7**	**- 1.6**	- 2.1	- 0.3	**- 1.2**	-	+ 0.1	- 0.1	** + 2.4**	**- 5.0**	+ 2.6
*P* value	**0.0036**	**0.0118**	0.1220	0.6486	**0.0002**		0.7179	0.9254	**0.0059**	**0.0041**	0.0606
**100G**											
Pre-treatment	96.2 ± 2.6	39.8 ± 0.9	14.0 ± 0.7	4.2 ± 0.4	5.7 ± 0.9	0.0 ± 0.0	13.7 ± 0.2	174.0 ± 1.9	64.1 ± 0.8	26.5 ± 0.5	9.3 ± 0.6
Treatment	61.4 ± 1.7	63.3 ± 0.7	14.7 ± 1.1	3.6 ± 0.2	4.8 ± 0.3	23.0 ± 1.2	12.1 ± 0.2	183.4 ± 1.8	44.0 ± 0.6	45.4 ± 0.9	10.6 ± 0.7
Treatment – Pre treatment	**- 34.8**	** + 23.5**	+ 0.7	- 0.6	- 0.9	** + 23.0**	**- 1.6**	** + 9.4**	**- 20.1**	** + 18.9**	+ 1.3
*P* value	**0.000039**	**0.000004**	0.3918	0.0602	0.1644	**0.000006**	**0.0004**	**0.0036**	**0.000004**	**0.000006**	0.0915
**Lbr**											
Pre-treatment	91.4 ± 1.5	32.9 ± 0.3	19.9 ± 0.5	5.9 ± 0.4	8.0 ± 0.2	0.0 ± 0.0	15.4 ± 0.3	173.6 ± 2.3	63.4 ± 0.3	22.8 ± 0.1	13.7 ± 0.3
Treatment	92.4 ± 1.9	36.1 ± 1.2	17.0 ± 0.3	4.0 ± 0.3	6.9 ± 0.7	0.0 ± 0.0	15.0 ± 0.6	171.5 ± 2.7	63.5 ± 0.7	24.7 ± 0.6	11.6 ± 0.4
Treatment – Pre treatment	+ 1.0	** + 3.2**	**- 2.9**	**- 1.9**	- 1.1	-	- 0.4	- 2.1	+ 0.1	** + 1.9**	**- 2.1**
*P* value	0.3524	**0.0121**	**0.0007**	**0.0017**	0.0582		0.4322	0.7846	0.7857	**0.0065**	**0.0019**
**Lbr-100G**											
Pre-treatment	89.6 ± 2.1	30.3 ± 0.9	27.1 ± 0.6	4.3 ± 0.5	5.4 ± 1.9	0.0 ± 0.0	14.9 ± 0.3	171.7 ± 3.1	60.9 ± 0.9	20.6 ± 0.6	18.5 ± 0.5
Treatment	74.0 ± 2.4	51.6 ± 0.2	22.8 ± 0.3	7.7 ± 0.6	10.4 ± 0.3	20.4 ± 0.3	13.2 ± 0.3	199.7 ± 3.1	49.9 ± 0.7	34.7 ± 0.6	15.4 ± 0.1
Treatment – Pre treatment	**- 15.6**	** + 21.3**	**- 4.3**	** + 3.4**	** + 5**	** + 20.4**	**- 1.7**	** + 28.0**	**- 11.0**	** + 14.1**	**- 3.1**
*P* value	**0.0011**	**0.000003**	**0.0004**	**0.0017**	**0.0152**	** < 0.000001**	**0.0022**	**0.0003**	**0.000085**	**0.000007**	**0.0004**

Data are means ± SD for the last 3 days of each treatment period. Delta corresponds the difference between treatment and pre-treatment. *P* value is calculated for the pairwise comparison of the last 3 days of treatment and pre-treatment periods within a reactor by unpaired *t*-test. CR, Control reactor; 100G, 100 mM glycerol; Lbr, *L. reuteri* PTA5_F13; Lbr-100G, *L. reuteri* PTA5_F13 and 100 mM glycerol

**Table 3:** Effect of glycerol supplementation and *L. reuteri* PTA5_F13 on the metabolic activity of chicken cecal microbiota during in vitro F3

Reactors	Mean metabolite concentration (mM) ± SD								Ratios of Total SCFAs (%)		
	**Acetate**	**Butyrate**	**Propionate**	**Succinate**	**Valerate**	**1,3 – PDO**	**Total BCFAs**	**Total metabolites**	**Acetate**	**Butyrate**	**Propionate**
**Control**											
Pre-treatment	83.9 ± 1.9	48.7 ± 0.5	11.7 ± 0.6	2.4 ± 1.5	9.7 ± 0.5	0.0 ± 0.0	15.0 ± 0.3	171.1 ± 4.0	58.1 ± 0.2	33.7 ± 0.6	8.1 ± 0.2
Treatment	76.5 ± 1.1	46.4 ± 0.6	10.7 ± 0.3	1.1 ± 0.1	9.0 ± 0.1	0.0 ± 0.0	16.6 ± 0.2	160.7 ± 1.6	57.2 ± 0.1	34.7 ± 0.3	8.0 ± 0.2
Treatment – Pre treatment	**- 7.4**	**- 2.2**	- 1.0	- 1.3	- 0.6		** + 1.6**	**- 10.8**	**- 0.8**	+ 0.9	0.1
*P* value	**0.0045**	**0.0092**	0.0609	0.1942	0.0769		**0.0009**	**0.0123**	**0.0091**	0.0611	0.6745
**100G**											
Pre-treatment	95.3 ± 3.0	44.9 ± 1.4	10.3 ± 1.4	0.7 ± 0.1	9.4 ± 0.2	0.0 ± 0.0	14.9 ± 0.4	175.8 ± 6.1	63.3 ± 0.5	29.8 ± 0.2	6.8 ± 0.7
Treatment	63.2 ± 1.0	83.1 ± 4.1	5.9 ± 0.8	2.1 ± 1.8	8.1 ± 0.8	22.3 ± 7.4	16.1 ± 0.6	178.6 ± 1.8	41.5 ± 1.0	54.5 ± 1.5	3.9 ± 0.5
Treatment – Pre treatment	**- 32.0**	** + 38.0**	**- 4.3**	+ 1.3	- 1.3	** + 22.3**	+ 1.0	+ 2.8	**- 21.8**	** + 24.7**	**- 2.9**
*P* value	**0.00006**	**0.0001**	**0.0107**	0.1114	0.0511	**0.0063**	0.0638	0.4868	**0.000005**	**0.00001**	**0.0055**
**Lbr**											
Pre-treatment	98.5 ± 2.6	43.7 ± 1.9	13.8 ± 0.5	1.0 ± 0.1	9.6 ± 0.5	0.0 ± 0.0	17.9 ± 0.5	184.6 ± 5.9	63.1 ± 0.3	28.0 ± 0.3	8.8 ± 0.1
Treatment	101.3 ± 0.9	36.3 ± 2.2	15.4 ± 1.7	1.0 ± 0.1	8.3 ± 0.3	0.0 ± 0.0	17.0 ± 0.7	179.8 ± 2.3	66.1 ± 0.2	23.7 ± 1.3	10.0 ± 1.2
Treatment – Pre treatment	+ 2.8	**- 7.3**	+ 1.6	-	**- 1.2**		- 0.8	- 4.8	** + 3.0**	** + 4.2**	+ 1.2
*P* value	0.1443	**0.0124**	0.1853	0.5772	**0.0220**		0.1619	0.2624	**0.0002**	**0.0050**	0.1422
**Lbr-100G**											
Pre-treatment	94.3 ± 3.7	46.3 ± 1.0	10.5 ± 2.4	3.3 ± 1.8	9.4 ± 0.8	0.0 ± 0.0	13.7 ± 2.5	177.7 ± 6.1	62.4 ± 1.9	30.6 ± 1.2	6.9 ± 1.5
Treatment	69.6 ± 4.8	86.7 ± 5.2	5.4 ± 0.8	5.5 ± 1.2	6.4 ± 1.1	61.4 ± 10.7	14.6 ± 3.3	187.9 ± 5.4	43.0 ± 3.0	53.6 ± 3.2	3.3 ± 0.4
Treatment – Pre treatment	**- 24.0**	** + 40.4**	**- 5.1**	+ 2.3	**- 3.1**	** + 61.4**	+ 0.5	+ 10.1	**- 19.3**	** + 22.9**	**- 3.6**
*P* value	**0.0021**	**0.0001**	**0.0256**	0.1529	**0.0170**	**0.0047**	0.8546	0.0986	**0.0007**	**0.00032**	**0.0182**

Data are means ± SD for the last 3 days of each treatment period. Delta corresponds the difference between treatment and pre-treatment. *P* value is calculated for the pairwise comparison of the last 3 days of treatment and pre-treatment periods within a reactor by unpaired *t*-test. CR, Control reactor; 100G, 100 mM glycerol; Lbr, *L. reuteri* PTA5_F13; Lbr-100G, *L. reuteri* PTA5_F13 and 100 mM glycerol

In conclusion, glycerol supplementation led to large increases in butyrate production and decreases in acetate production in all three models, along with 1,3-PDO accumulation.

### Impact of glycerol on chicken cecal microbiota composition

Prior to treatment, total bacteria as measured by qPCR in the CR of each model were 10.3 ± 0.1, 10.7 ± 0.0 and 11.3 ± 0.0 log gene copies in F1, F2 and F3, respectively (Tables 4, 5 and 6). Upon glycerol supplementation, total bacteria remained unchanged in all three models compared to CR for F1 or compared to pre-treatment for F2 and F3 (Figs. S4, S5 and S6); however, glycerol impacted the concentration of specific bacterial groups tested with qPCR. Treatment with 50G in F1 resulted in a significant (*p* < 0.05) decrease of *Enterobacteriaceae* spp. (- 0.6 log gene copies), while 100G resulted in a significant increase in *Bacteroidetes* (+ 0.7 log gene copies) compared to CR (Table 4). These two taxa exhibited microbiota-dependent responses to glycerol when comparing their concentration after treatment with pre-treatment in F2 and F3. For *Bacteroidetes*, an increase in F3 (+ 1.0 log gene copies) (Table 6) was observed, compared to a small decrease in F2 (- 0.4 log gene copies) (Table 5). For *Enterobacteriaceae*, 100G resulted in a significant decrease in F2 (-0.7 log gene copies) compared to pre-treatment, but not in F3, where *Enterobacteriaceae* was present at high concentrations between log 9.2 to log 10.2 during the pre-treatment period.

**Table 4:** Log 16S rRNA gene copy numbers of specific bacterial groups for the different experimental conditions of modelled cecal microbiota F1, enumerated by qPCR

	**Log10 16S rRNA gene copies of taxon/mL (mean ± SD)**						
	**Total bacteria**	**Firmicutes**	***Ruminococcaceae***	***Lactobacillus-Leuconostoc-Pediococcus* spp.**	**Bacteroidetes**	***Enterobacteriaceae***	***Bifidobacteriaceae***
**CR**							
Treatment	10.3 ± 0.1	10.1 ± 0.1	10.1 ± 0.1	6.6 ± 0.4	9.1 ± 0.1	7.5 ± 0.2	7.7 ± 0.1
**50G**							
Treatment	10.3 ± 0.1	10.1 ± 0.0	10.1 ± 0.1	6.9 ± 0.3	9.2 ± 0.1	6.9 ± 0.1	7.4 ± 0.0
Delta with CR	+ 0.03	+ 0.07	+ 0.07	+ 0.3	+ 0.1	**- 0.6**	- 0.3
*P* value with CR	0.7776	0.2302	0.4216	0.3673	0.3586	**0.0158**	0.1161
**100G**							
Treatment	10.4 ± 0.1	10.1 ± 0.1	10.1 ± 0.1	7.2 ± 0.1	9.8 ± 0.2	7.8 ± 0.0	7.0 ± 0.5
Delta with CR	+ 0.2	0.0	0.0	+ 0.5	** + 0.7**	+ 0.3	-0.6
*P* value with CR	0.2061	> 0.9999	> 0.9999	0.1462	**0.0031**	0.1144	0.0859

Data are means ± SD for the last 3 days of each treatment period. Significant differences were calculated as compared to Control reactor (*P* < 0.05) by unpaired *t*-test. CR, Control reactor; 50G, 10 mM glycerol; 100G, 100 mM glycerol

**Table 5:** Log 16S rRNA gene copy numbers of specific bacterial groups for the different experimental conditions of modelled cecal microbiota F2, enumerated by qPCR

	**Log10 16S rRNA gene copies of taxon/mL (mean ± SD)**							
	**Total bacteria**	**Firmicutes**	***Ruminococcaceae***	***Lactobacillus-Leuconostoc-Pediococcus* spp.**	**Bacteroidetes**	***Enterobacteriaceae***	***Bifidobacteriaceae***	***L. reuteri*** **PTA5_F13**
**CR**								
Pre-treatment	10.7 ± 0.0	9.9 ± 0.1	9.8 ± 0.0	6.4 ± 0.1	9.9 ± 0.1	8.5 ± 0.3	7.2 ± 0.1	BDL
Treatment	10.6 ± 0.1	9.8 ± 0.1	9.8 ± 0.1	6.1 ± 0.0	9.9 ± 0.0	8.2 ± 0.2	7.2 ± 0.1	BDL
*P* value	0.116	0.251	0.374	** < 0.001**	0.116	0.284	0.230	-
**100G**								
Pre-treatment	10.8 ± 0.1	9.9 ± 0.1	9.9 ± 0.1	7.8 ± 0.2	8.9 ± 0.1	8.1 ± 0.1	8.4 ± 0.2	BDL
Treatment	10.8 ± 0.1	10.0 ± 0.0	10.0 ± 0.0	8.1 ± 0.3	8.5 ± 0.2	7.4 ± 0.2	8.3 ± 0.1	BDL
*P* value	0.519	0.116	0.116	0.187	**0.013**	**0.003**	0.348	-
**Lbr**								
Pre-treatment	10.9 ± 0.1	9.8 ± 0.0	9.8 ± 0.1	7.3 ± 0.3	9.5 ± 0.2	7.4 ± 0.1	7.7 ± 0.2	BDL
Treatment	10.8 ± 0.3	9.9 ± 0.1	9.9 ± 0.0	8.9 ± 0.1	9.2 ± 0.1	7.3 ± 0.2	8.2 ± 0.1	7.6 ± 0.1
*P* value	0.442	**0.007**	0.116	** < 0.001**	0.193	0.435	**0.010**	** < 0.001**
**Lbr-100G**								
Pre-treatment	11.0 ± 0.1	9.8 ± 0.0	9.8 ± 0.1	6.4 ± 0.2	10.0 ± 0.1	8.1 ± 0.1	7.3 ± 0.0	BDL
Treatment	11.1 ± 0.1	9.8 ± 0.1	9.8 ± 0.1	8.9 ± 0.1	9.9 ± 0.1	6.9 ± 0.1	7.2 ± 0.1	7.7 ± 0.1
*P* value	0.374	0.374	0.230	** < 0.001**	0.319	** < 0.001**	0.116	** < 0.001**

Data are means ± SD for the last 3 days of each treatment period. *P* value is calculated for the pairwise comparison of the last 3 days of treatment and pre-treatment periods within a reactor by unpaired *t*-test. BDL, below the detection limit (4.8 log gene copies, *L. reuteri* PTA5_F13). CR, Control reactor; 100G, 100 mM glycerol; Lbr, *L. reuteri* PTA5_F13; Lbr-100G, *L. reuteri* PTA5_F13 and 100 mM glycerol

**Table 6:** Log 16S rRNA gene copy numbers of specific bacterial groups for the different experimental conditions of modelled cecal microbiota F3, enumerated by qPCR

	**Log10 16S rRNA gene copies of taxon/mL (mean ± SD)**							
	**Total bacteria**	**Firmicutes**	***Ruminococcaceae***	***Lactobacillus-Leuconostoc-Pediococcus* spp.**	**Bacteroidetes**	***Enterobacteriaceae***	***Bifidobacteriaceae***	***L. reuteri*** **PTA5_F13**
**CR**								
Pre-treatment	11.3 ± 0.0	10.5 ± 0.1	10.6 ± 0.1	6.9 ± 0.1	5.9 ± 0.2	9.4 ± 0.4	4.9 ± 0.0	BDL
Treatment	11.2 ± 0.1	10.4 ± 0.1	10.1 ± 0.1	7.5 ± 0.1	5.6 ± 0.0	8.7 ± 0.1	4.7 ± 0.1	BDL
*P* value	0.413	0.124	**0.005**	**0.003**	**0.039**	**0.007**	**0.005**	-
**100G**								
Pre-treatment	11.2 ± 0.1	10.5 ± 0.1	10.2 ± 0.1	6.5 ± 0.2	5.7 ± 0.1	9.2 ± 0.0	4.9 ± 0.1	BDL
Treatment	11.2 ± 0.1	10.5 ± 0.2	10.2 ± 0.2	6.2 ± 0.1	6.7 ± 0.1	8.9 ± 0.3	4.9 ± 0.0	BDL
*P* value	0.529	0.800	0.937	0.186	**0.001**	0.235	0.644	-
**Lbr**								
Pre-treatment	11.1 ± 0.2	10.5 ± 0.0	10.2 ± 0.1	6.6 ± 0.5	5.5 ± 0.2	9.4 ± 0.7	4.8 ± 0.3	BDL
Treatment	11.1 ± 0.1	10.4 ± 0.1	10.2 ± 0.0	6.3 ± 0.2	5.6 ± 0.1	9.8 ± 0.2	5.0 ± 0.1	5.1 ± 0.5
*P* value	0.923	0.080	0.462	0.369	0.836	0.463	0.349	**0.001**
**Lbr-100G**								
Pre-treatment	11.2 ± 0.3	10.5 ± 0.2	10.4 ± 0.1	6.8 ± 0.6	6.0 ± 0.0	10.2 ± 0.3	4.9 ± 0.1	BDL
Treatment	11.2 ± 0.1	10.6 ± 0.1	10.3 ± 0.2	8.0 ± 0.3	6.4 ± 0.1	9.4 ± 0.3	4.8 ± 0.1	6.1 ± 0.9
*P* value	0.825	0.583	0.766	0.052	**0.002**	0.052	0.644	**0.002**

Data are means ± SD for the last 3 days of each treatment period. *P* value is calculated for the pairwise comparison of the last 3 days of treatment and pre-treatment periods within a reactor by unpaired *t*-test. BDL, below the detection limit (4.8 log gene copies, *L. reuteri* PTA5_F13). CR, Control reactor; 100G, 100 mM glycerol; Lbr, *L. reuteri* PTA5_F13; Lbr-100G, *L. reuteri* PTA5_F13 and 100 mM glycerol

**Fig. 2:**
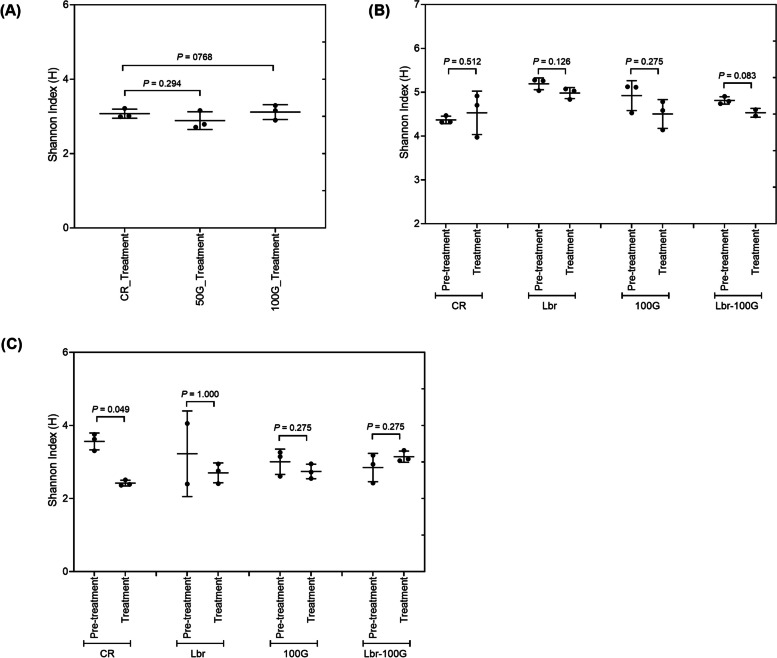
Alpha diversity measured by Shannon index in microbiota from three independent in vitro fermentations under different conditions. F1 (**A**), F2 (**B**) and F3 (**C**). Values are mean of results ± standard deviation of the last 3 days of fermentation. CR, Control reactor; 50G, 50 mM glycerol; 100G, 100 mM glycerol; Lbr, L. reuteri PTA5_F13; Lbr-100G, L. reuteri PTA5_F13 and 100 mM glycerol

The microbial composition profile and diversity were assessed in all reactors by 16S rRNA metabarcoding. Community diversity measured by Shannon-index was similar in all reactors, and between pre-treatment and treatment within a model and independently of glycerol addition (Fig. 2).

Few taxa responded to glycerol treatment among the three modelled microbiota, as measured by DESeq2 analysis. In CR of F1, F2 and F3, one or two taxa significantly decreased or increased between pre-treatment and treatment phases, supporting the stability of the models (Figs. S7 and S8). In F1, supplementation with 50G and 100G significantly increased *Lactobacillus* ASVs (fold change: 17.6), compared to CR. In F2, significant increases in *Anaerobutyricum hallii*_group ASVs (fold-change: 4.4 (ASV032) and 4.7 (ASV019)), *Faecalibacterium* UBA1819 ASV045 (fold-change: 3.0), *Enterococcus* ASVs (fold-change: 2.4 (ASV035) and 8.2 (ASV044)) and *Monoglobus* ASV067 (fold-change: 2.9) were observed when supplemented with 100G. In F3, only *Enterococcus* ASV018 was enriched (fold-change: 7.1) during 100G treatment.

Overall, supplementation with glycerol alone did not show large effects on the community diversity and microbiota composition, but it did strongly enhanced butyrate production while inhibiting *Enterobacteriaceae* in F1 (50G) and F2 (100G).

### Impact of *L. reuteri* alone and in combination with glycerol on chicken cecal microbiota metabolites 

The capacity of Lbr to produce reuterin from glycerol and its impacts in the modelled chicken cecal microbiota was tested for 8 days in F2 and F3. Sole addition of Lbr did not affect the microbiota metabolism compared with the corresponding pre-treatment periods, as indicated by stable concentrations of total metabolites, SCFAs and BCFAs (Tables 2 and 3). The combination of *L. reuteri* PTA5_F13 and 100 mM glycerol (Lbr-100G) resulted in similar effects to 100G treatment, with significantly increased butyrate concentrations (+ 21.3 mM and + 40.4 mM in F2 and F3, respectively) and relative ratios to total SCFA (+ 14.1% and + 22.9%), and decreased acetate concentrations (-15.6 mM and -24 mM) and ratio (-11.0% and -19.3%), compared to pre-treatment period. Glycerol had no effect on detected amounts of the intermediate products succinate and valerate. Furthermore, 1,3-PDO was only produced in the presence of glycerol (20.4 mM and 61.4 mM in F2 and F3, respectively), while 3-HPA remained below the detection limit of 2.0 ± 0.7 mM.

### Colonization and impact of *L. reuteri* alone and combined with glycerol on chicken cecal microbiota composition

Walter et al. previously developed and validated qPCR primers targeting the glycerol/diol dehydratase gene (*pduC*), present at one copy per cell in the reuterin-producing *L. reuteri* strains [23]. Hence, these primers to monitor the colonization of reuterin-producing *L. reuteri* PTA5 qPCR analysis [23]. Reuterin-producing *L. reuteri* was below detection levels (DL of 4.8 log gene copies) in CR and TRs reactors during the pretreatment period for both F2 and F3. Upon daily spiking (final concentration of 10^7^ cells/mL) for 8 days, stable gene copy numbers of reuterin-producing *L. reuteri* of 7.7 ± 0.1 and 6.1 ± 0.1 log gene copies (cells)/mL were measured in spiked test reactors of F2 and F3, respectively (Tables 5 and 6). Lbr treatment was associated with specific quantitative (by qPCR) compositional changes in the microbiota. In F2, an increase of *Lactobacillus*-*Leuconostoc*-*Pediococcus* spp. (+ 1.6 log gene copies/mL to 8.9 log gene copies/mL) and *Bifidobacteriaceae* (+ 0.5 log gene copies/mL) (Table 5), while no significant effect was detected during F3 for any of the tested bacterial groups (Table 6) compared to the corresponding reactor pre-treatment period.

The microbial community diversity of F2 and F3 as measured by 16S rRNA metabarcoding and Shannon index was not affected by Lbr treatment compared to the pre-treatment period (Fig. 2). In F2, specific bacterial taxa were enriched during Lbr treatment, including *Clostridium innocuum* ASV075 (fold-change: 2.1), *Lactobacillus* ASV016 (fold-change: 13.8), *Monoglobus* ASV067 (fold-change: 7.4) and *Faecalibacterium* UBA1819 ASV045 (fold-change: 3.5) (Fig. S7), while no taxa were enriched during the same treatment in F3 (Fig. S8). Further, *Escherichia-Shigella* ASV021 decreased in Lbr (fold change: 2.9); the decrease was even stronger by the combined treatment Lbr-100G (fold change: 4.9) in F2 (Fig. S7). In both F2 and F3 the combined treatment Lbr-100G induced an increase of *Lactobacillus*-*Leuconostoc*-*Pediococcus* spp. (+ 2.5 log gene copies/mL, *P* < 0.001, and + 1.2 log gene copies/mL, *P* = 0.052, in F2 and F3, respectively) and a significant decrease of *Enterobacteriaceae* (-1.2 log gene copies/mL, *P* < 0.001, and -0.8 log gene copies/mL, *P* = 0.052, in F2 and F3, respectively) compared to the pre-treatment period (Tables 5 and 6). Similarly, when compared to single treatments, the combined treatment did not change alpha diversity in F2 or F3 when compared to the pre-treatment period (Fig. 2). In F2, a significant enrichment of the relative abundance of *Alistipes* ASV100 (fold-change: 4.7), *Anaerobutyricum hallii*_group ASVs (fold-change: 4.4 (ASV032) and 10.86 (ASV019)), *Faecalibacterium* UBA1819 ASV045 (fold-change: 2.1) and *Lactobacillus* ASV016 (fold-change: 9.1) was observed with DESeq2 analysis during Lbr-100G treatment (Fig. S8). However, in F3 no significant enrichment of bacterial taxa was detected during Lbr-100G treatment.

## Discussion

In this study, we investigated the effect of glycerol and reuterin-producing *L. reuteri* supplementation alone and in combination on modelled chicken gut microbiota composition and activity using a newly developed chicken cecal PolyfermS model inoculated with immobilized microbiota [30]. Our data showed for the first time a pronounced butyrogenic effect of glycerol associated with a specific stimulation of butyrate-producing taxa in the chicken cecal microbiota. Concurrently, a reduction of *Enterobacteriaceae* by glycerol was observed in F1 and F2 models, while the combined treatment Lbr-100G showed inhibition in all three models. Interestingly, the F3 model showed strong colonization of *Enterobacteriaceae* during the pre-treatment period, with levels in the range of log 9.2 to log 10.2 gene copies/mL, which were approximately 2 to 3 log higher than in F1 and F2. *Enterobacteriaceae* are known to be highly sensitive to the antimicrobial effect of reuterin [18, 30] and it was proposed that acrolein, not 3-HPA, is the active compound responsible for the main antimicrobial activity attributed to reuterin [20, 31]. However, reuterin and acrolein could not be measured in the reactor effluent, likely due to the high reactivity of both compounds with amino- or sulfhydryl groups in the medium or with bacteria [32, 33]. Similar to our study, Cleusix et al. [33] observed increased numbers of the *Lactobacillus*-*Enterococcus* group and decreased *E. coli*, but no robust effect on butyrate production upon the addition of 100 mM glycerol, alone or together with reuterin-producing *L. reuteri* of human origin in an adult PolyFermS colonic model. This was attributed to in situ reuterin production because 1,3-PDO, a typical product of glycerol fermentation, was detected.

The addition of glycerol modulated metabolite production and SCFA ratios, with a large increase of butyrate at the expense of acetate in the three modelled chicken cecal microbiota. Glycerol can be metabolized by various bacteria to form acetate, butyrate, lactate, succinate, ethanol,* n*-butanol and 2,3-butanediol via the oxidative branch [34, 35]. Alternatively, glycerol can be reduced to 1,3-PDO, a product that is not found in anaerobic conversions of other organic substances [36]. Here, we observed a marked increase in 1,3-PDO when glycerol was supplemented independently of spiking *L. reuteri* PTA5_F13, indicating that 1,3-PDO was produced by other cecal taxa. A number of intestinal taxa have the ability to convert glycerol into 1,3-PDO, including *Klebsiella*, *Enterobacter*, *Citrobacter*, *Clostridium*, and *Eubacterium* [22, 37–39]. Interestingly, among the 1,3-PDO producers, *A. hallii* (renamed from *Eubacterium hallii*), *Clostridium butyricum* and *Clostridium perfringens* can also produce butyrate as a by-product during glycerol fermentation [21, 36].

Butyrate is commonly used as a feed additive in chicken breeding [34, 40, 41]. Butyrate has a wide range of cellular functions, including anti-inflammatory effects, promotion of gut tissue development, reinforcement of the epithelium barrier and pathogen control, which may explain the observed increase in the productive performance of chicken [42–45]. Hence, endogenous (microbial) production of butyrate from glycerol in the cecum may be an effective approach for promoting in situ butyrate production.

Administering *L. reuteri* to poultry has been reported to have beneficial effects on poultry performance and health [14, 15, 46]. *L. reuteri* can form biofilms in the chicken crop, which persists throughout the host’s life [9, 47, 48]. From the crop biofilm, bacteria are constantly shed and transferred to the lower GIT; thus, *L. reuteri* is also commonly encountered in the cecum and colon of the chicken [11]. Here, we used daily spiking of chicken-isolated *L. reuteri* PTA5_F13 to mimic the continuous shedding of *L. reuteri* from the chicken crop. A stable microbiota-dependent colonization of reuterin-producing strain *L. reuteri* PTA5_F13 was demonstrated upon spiking in the three models, but the treatment alone did not induce change the microbiota composition and metabolic activity.

## Conclusions

Using the continuous chicken cecal microbiota PolyFermS model we showed that glycerol induced a stable and reproducible butyrogenic activity and a reduction of *Enterobacteriaceae* upon glycerol supplementation and *L. reuteri* supplementation at very high *Enterobacteriaceae* concentrations. Only minor effects on a limited number of taxa of the chicken microbiota were measured for individual and combined treatments. We speculate that the reported benefits of glycerol such as improving body weight gain and feed conversion efficiency in chickens may be partly due to stimulating endogenous butyrate production while preserving the composition and activity of commensals in the chicken microbiota. Further, in vivo studies are needed to evaluate the potential use of glycerol in poultry nutrition and inhibition of enteropathogenic taxa belonging to *Enterobacteriaceae*.

## Methods 

### *L. reuteri* strain and growth conditions 

*L. reuteri* (strain PTA5_F13, in short Lbr; culture collection of the Laboratory of Food Biotechnology, ETH Zürich, Zürich, Switzerland) was previously isolated from feces of a healthy chicken and selected for this study for its high reuterin production [24]. The strain was reactivated from frozen glycerol stock (30% vol/vol, kept at -80 °C) and routinely cultured under anaerobic conditions supplied by a gas package (AnaeroGen, Thermo Fisher Diagnostics AG, Pratteln, Switzerland) in anaerobic jars for 16 h at 37 °C in de Man, Rogosa and Sharpe medium (MRS, Biolife, Milan, Italy). For daily spiking of reactors (TR2 and TR3), *L. reuteri* cultures (30 mL set at a concentration of 10^9^ CFU/mL) were harvested by centrifugation at 3000 × *g* for 3 min, the supernatant was discarded, and the bacterial pellet was washed once with 0.1 M phosphate-buffered saline (PBS) and resuspended in PBS (3 mL) to a concentration of 10^7^ CFU/reactor upon spiking. Viable cell counts were measured by plating on MRS agar plates after incubating anaerobically at 37 °C for 24 h.

### Nutritive medium for in vitro chicken cecal microbiota fermentation 

The description of baseline data of the three in vitro fermentation models (F1, F2 and F3) that were used to develop and validate the chicken cecal PolyFermS model were previously presented in detail [30]. The mVL-1 nutritive medium was used to cultivate cecal microbiota in the PolyFermS chicken model in F1 [30]. This medium was previously developed to mimic the chicken cecal microbiota profile and activity, and contains (g/L in distilled water): beef extract (2.4), yeast extract (5.0), maltodextrin (2.5), tryptose (10), L-cysteine HCl (0.8), NaCl (5.0), mucin (2.0), uric acid (0.7), Tween 80 (1 mL), bile salts (0.4), KH_2_PO_4_ (0.5), NaHCO_3_ (1.5), KCl (4.5), MgSO_4_ anhydrous (0.6), CaCl_2_·2H_2_O (0.1), MnCl_2_·4H_2_O (0.2), FeSO_4_·7H_2_O (0.005), and hemin (0.05). The mVL-3 nutritive medium, which is similar in composition to mVL-1 but enriched with fructooligosaccharides (FOS) (2.5 g/L) and citrus pectin (2.5 g/L), was used in F2 and F3 [30]. When required, glycerol (50 or 100 mM) was added to the nutritive medium. All constituents except FOS were dissolved in distilled water, and the medium was adjusted to pH 6.0 using 2.5 M HCl and autoclaved at 121 °C for 20 min. After sterilizing and cooling to 4 °C, 2.5 g/L of filter-sterilized FOS (Cosucra Group, Warcoing, Belgium) and 1 mL of a filter-sterilized (0.2 μm pore-size) vitamin solution [49] were added to the medium. All components were purchased from Sigma-Aldrich Chemie (Buchs, Switzerland), except bile salts (Oxoid AG), yeast extract (Merck, Darmstadt, Germany), NaHCO_3_ (Fisher Scientific, Pittsburgh, USA), NaCl and KH_2_PO_4_ (VWR International AG, Dietikon, Switzerland), MgSO_4_ anhydrous (Acros Organics, Geel, Belgium) and MnCl_2_·4H_2_O (Fluka, Buchs, Switzerland).

### Experimental setup and fermentation procedure 

**Fig. 3:**
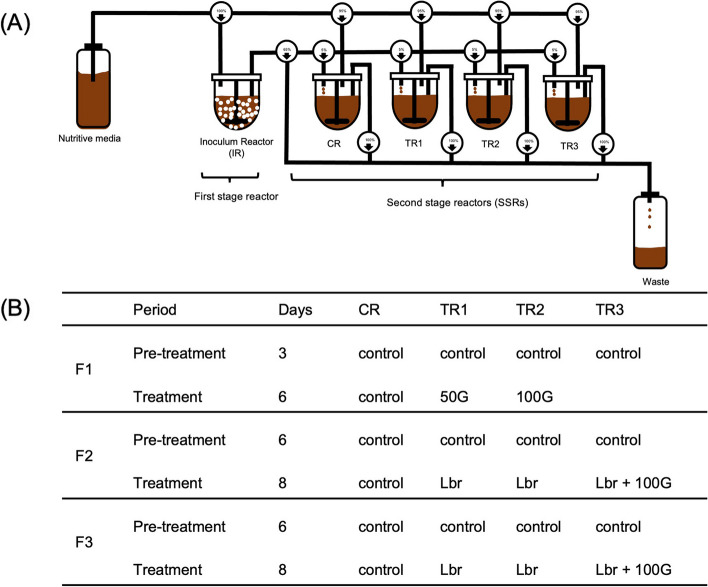
Experimental set-up of the PolyFermS chicken cecal in vitro models. **A** Experimental set-up and conditions tested in the PolyFermS model mimicking the chicken cecal microbiota. Control reactor (CR) and test reactors (TR) were continuously inoculated with 5% fermentation effluent from the inoculum reactor (IR) and 95% nutritive media. **B** Experimental conditions for three independent fermentations (F1, F2 and F3) during the pre-treatment and treatment periods. *L. reuteri* PTA5_F13 (Lbr) was added daily for 8 days in F2 and F3 to reach a concentration of 10^7^ CFU/mL. 50G, 50 mM glycerol; 100G 100 mM glycerol

Three independent in vitro chicken cecal fermentations (F1, F2, and F3) inoculated with immobilized cecal microbiota from three different donor animals were carried out as depicted in Fig. 3. Details on establishing and stabilizing the three models operated in conditions mimicking the chicken cecum were presented in detail in our previous paper (specifically: F1A, F2 and F3) [30]. Briefly, for each fermentation, the microbiota of a freshly-collected cecal sample from a 21-day-old Cobb-500 broiler chicken was sampled and immobilized in 1–2 mm diameter gellan-xanthan gum gel beads within 2 h of collection. All steps from collection of cecal content to reactor inoculation were carried out in anaerobic conditions, using a gas package and an anaerobic chamber [30]. The cecal microbiota beads (60 mL) were immediately transferred to a 0.5 L fully controlled bioreactor (Multifors; Infors AG) containing 140 mL mVL-3 medium and colonized during two consecutive batch fermentations of 20 and 6 h, respectively, before switching to continuous mode. The model was operated at conditions selected to mimic the chicken cecum: pH 6.0, stirring at 180 rpm, 41 °C, mean retention time of 24 h, and continuous CO_2_ headspace flushing. Each model consisted of an inoculum reactor (IR) inoculated with 30% (v/v) cecal microbiota-colonized beads with a total fermentation volume of 200 mL. After an initial stabilization period of 16 to 20 days, the IR effluent was used to continuously inoculate (5%, v/v) parallel second-stage reactors with the same microbiota. Second-stage reactors were additionally supplied with 95% (v/v) sterile fresh nutritive medium and further stabilized for a period of 3 days (F1) or 6 days (F2 and F3) before starting treatments. This experimental setup allowed simultaneous testing of different treatments applied in three treatment reactors (TRs) and comparison to an untreated control reactor (CR).

Model F1 was used to test the effects of two concentrations of glycerol (final concentration of 50 [50G] and 100 mM [100G]) on microbiota population and metabolite production, whereas models F2 and F3 were used to test the effect of glycerol (100 mM [100G]) and *L. reuteri* PTA5_F13 (added daily at a final concentration of 10^7^ CFU/mL [Lbr]) alone or combined (100 mM [Lbr-100G]) (Fig. 3B). During the treatment period, CR was fed with medium without glycerol, and supplemented with 2 mL (equivalent inoculum volume) of 0.1 M PBS when *L. reuteri* was tested in TRs (F2 and F3). Each treatment was performed for 6 or 8 days until stability, which was defined by less than 10% variations in the daily metabolite concentrations, was reached. Reactor effluents were sampled daily and centrifuged (10 min at 14′000 × *g* at 4 °C). Bacterial pellet and supernatant were stored at -80 °C and -20 °C for DNA extraction and metabolite analysis, respectively.

### Metabolite analysis

SCFAs (acetate, propionate, butyrate, valerate), branched-chain fatty acids (BCFAs: iso-butyrate and iso-valerate), intermediate metabolites (succinate, formate, lactate), 3-HPA and 1,3 PDO were measured during the last three days of the pre-treatment and treatment periods by high-performance liquid chromatography with refractive index detector (HPLC-IR), as presented previously [30], and expressed as millimole per liter effluent (mM).

### DNA extraction

Total genomic DNA was extracted from the cell pellet of 2 mL of effluent using the FastDNA® SPIN Kit for Soil (MP Biomedicals, Illkirch Cedex, France) and a final elution volume of 100 µL, according to the manufacturer’s instructions. DNA concentrations and quality were determined by absorbance measured at 260 nm using a Nanodrop® ND-1000 Spectrophotometer (Wiltec AG, Littau, Switzerland). Samples were stored at -20 °C before qPCR and 16S rRNA metabarcoding analysis.

### Quantitative PCR analysis

Quantitative PCR (qPCR) analysis was performed using a Roche LightCycler 480 II (Roche Diagnostics AG, Rotkreuz, Switzerland). Specific primers (Table S1) targeting selected bacterial groups of chicken gut microbiota were used at a final concentration of 200 nM. Amplification conditions and quantification were carried out as previously described [30]. Briefly, the diluted DNA (1 μL) was used for amplification in duplicate in a 20 μL reaction solution, containing 10 μL of SensiFAST SYBR No-ROX Kit (Bioline, Luckenwalde, Germany) and 200 nM of each primer. Reactions were denaturalized in LightCycler 480 Multiwell plate 96 (Roche Diagnostics AG) at 95 °C for 3 min, followed by 45 cycles at 95 °C for 5 s and 60 °C for 30 s. qPCR data were analyzed using the LightCycler® 480 Software 1.5.1.

### Microbial profiling with 16S rRNA metabarcoding 

16S rRNA amplicon sequencing of bacterial communities in effluents were conducted using an Illumina Miseq platform (Genetic Diversity Centre, ETH Zurich). Effluent samples were amplified and sequenced using universal primers targeting the V3 (for F1 and F2) or V4 (for F3) region of the 16S rRNA (Table S1). Preparation of the sequence library and sequencing steps were conducted as previously described [30].

Quantitative Insights Into Microbial Ecology 2 (QIIME 2) version 2020.8 was used for analysis of the sequence data [50]. The sequences were imported into QIIME 2 using a Casava 1.8 single-end demultiplexed format. DADA2, a pooled-sample chimera filtering method, was used to denoise the sequences [51]. VSEARCH was used to identify non-16S rRNA genes, chimeric sequences, and open reference clustering of amplicon sequence variants (ASVs) [52]. All ASVs were aligned de novo using MAFFT and used to construct a phylogenetic tree with FastTree 2 (via q2­phylogeny) [53, 54]. Taxonomy was assigned to ASVs using a pre-trained scikit­learn naïve Bayes classifier referencing SILVA database (v. 138) with a 99% identity threshold from 388F/518R (V3) or 515F/806R (V4) region of sequences [55–57]. Feature tables representing the ASV counts for each sample were made in the HDF5­based biological observation matrix (BIOM) format version.

### Statistical analysis

GraphPad Prism version 8.0 (GraphPad Software, San Diego, CA) was used to visualize the data. All statistical analyses were performed with IBM SPSS 28.0.0 (IBM SPSS Statistics for Windows, NY, USA), and* P* values less than 0.05 were considered significant. qPCR (log10 transformed) and HPLC-IR data were expressed as mean ± standard deviation of the last 3 days of each fermentation period, corresponding to stable metabolite production as indicated above. Statistical analysis was performed by pairwise comparison between the last 3 days of treatment between CR and TRs during the same period (F1) or between the pre-treatment and treatment periods of the same reactor (F2 and F3) by *t-*test. Comparison within a reactor was selected for F2 and F3 to account for differences in metabolite composition, qPCR data and 16S data observed between reactors during the pre-treatment periods [30]. The DESeq2 method was used to test significant differences in taxa abundance between the microbiota, using the same type of comparison as described above [58].

### Supplementary Information

**Additional file 1: Table S1.** Primers used for the detection of bacterial groups by qPCR and for the PCR amplification step of 16S rRNA amplicon Illumina sequencing. **Figure S1.** Daily metabolite concentrations in the effluent of control and treatment reactors during F1 measured by HPLC-IR: control reactor) (A), 50 mM glycerol (50G) supplementation (B), and 100 mM glycerol (100G) supplementation (C). Lactate was below detection limit. **Figure S2.** Daily metabolite concentrations in the effluent of control and treatment reactors during F2 measured by HPLC-IR: control reactor) (A), *L. reuteri* (Lbr) supplementation (B), 100 mM glycerol (100G) supplementation (C), and *L. reuteri* and 100 mM glycerol (Lbr-100G) supplementation (D). Lactate was below detection limit. **Figure S3.** Daily metabolite concentrations in the effluent of control and treatment reactors during F3 measured by HPLC-IR: control reactor) (A), *L. reuteri* (Lbr) supplementation (B), 100 mM glycerol (100G) supplementation (C), and *L. reuteri* and 100 mM glycerol (Lbr-100G) supplementation (D). Lactate was below detection limit. **Figure S4.** Daily quantification of key bacterial populations in the effluent of control and treatment reactors during F1 measured by qPCR: control reactor) (A), 50 mM glycerol (50G) supplementation (B), and 100 mM glycerol (100G) supplementation (C). **Figure S5.** Daily quantification of key bacterial populations in the effluent of control and treatment reactors during F2 measured by qPCR: control reactor) (A), *L. reuteri (*Lbr) supplementation (B) 100 mM glycerol (100G) supplementation (C), and *L. reuteri* and 100 mM glycerol (Lbr-100G) supplementation (D). **Figure S6.** Daily quantification of key bacterial populations in the effluent of control and treatment reactors during F3 measured by qPCR: control reactor) (A), *L. reuteri (*Lbr) supplementation (B) 100 mM glycerol (100G) supplementation (C), and *L. reuteri* and 100 mM glycerol (Lbr-100G) supplementation (D). **Figure S7.** Genus taxa with differential relative abundance in reactor effluent during treatment compared to pre-treatment (DeSeq2 analysis) in modelled cecal microbiota F2. Taxa are ordered according to log2 fold-change. Each ASV affected for a given genus is indicated with a dot. Dots are coloured according to phylum with the color code in the graph legend. Only taxa with more than 2 log2 fold-change and which are significantly differentially abundant (LRT, *P* ≤ 0.05) are shown. CR, Control reactor; 100G, 100 mM glycerol ; Lbr, *L. reuteri* PTA5_F13; Lbr-100G*, L. reuteri* PTA5_F13 and 100 mM glycerol. **Figure S8.** Genus taxa with differential relative abundance in reactor effluent during pre-treatment compared with treatment (DeSeq2 analysis) in modelled cecal microbiota F3. Taxa are ordered according to log2 fold-change. Each ASV affected for a given genus is indicated with a dot. Dots are coloured according to their phylum with the color code in the graph legend. Only taxa with at least 2 log2 fold-change and which are significantly differentially abundant (LRT, *P* ≤ 0.05) are shown. CR, Control reactor; 100G, 100 mM glycerol ; Lbr, *L. reuteri* PTA5_F13; Lbr-100G, *L. reuteri* PTA5_F13 and 100 mM glycerol.

## Data Availability

The sequence data reported in this paper have been deposited in the European Nucleotide Archive database (primary accession no. PRJEB56746).

## Abbreviations

qPCR: Quantitative polymerase chain reaction
GIT: Gastrointestinal tract
SCFA: Short-chain fatty acids
3-HPA: 3-Hydroxypropionaldehyde
1,3-PDO: 1,3-Propanediol
PCR: Polymerase chain reaction
HPLC: High-performance liquid chromatography
pduC: Glycerol/diol dehydratase
buk: Butyrate kinase
PBS: Phosphate- buffered Saline
FOS: Fructooligosaccharides
IR: Inoculum reactor
CR: Control reactor
TR: Test reactors
BCFA: Branched-chain fatty acids
QIIME2: Quantitative Insights Into Microbial Ecology 2
ASV: Amplicon sequence variants
PCA: Principal component analysis
NMDS: Non-metric multidimensional scaling
16S rDNA: 16S ribosomal deoxyribonucleic acid

## References

[1] Huang P, Zhang Y, Xiao K, Jiang F, Wang H, Tang D (2018). The chicken gut metagenome and the modulatory effects of plant-derived benzylisoquinoline alkaloids 06 Biological Sciences 0605 Microbiology. Microbiome..

[2] Rychlik I (2020). Composition and function of chicken gut microbiota. Animals..

[3] Rinttilä T, Apajalahti J (2013). Intestinal microbiota and metabolites — Implications for broiler chicken health and performance. J Appl Poult Res..

[4] Sergeant MJ, Constantinidou C, Cogan TA, Bedford MR, Penn CW, Pallen MJ. Extensive microbial and functional diversity within the chicken cecal microbiome. PLoS One. 2014;9:e91941. 10.1371/journal.pone.0091941 PMC3962364 24657972

[5] Shang Y, Kumar S, Oakley B, Kim WK. Chicken gut microbiota: Importance and detection technology. Front Vet Sci. 2018;5:254. 10.3389/fvets.2018.00254 PMC6206279 30406117

[6] EFSA (European Food Safety Authority). The European Union summary report on antimicrobial resistance in zoonotic and indicator bacteria from humans, animals and food in 2016. EFSA J. 2018;16:e05182 10.2903/j.efsa.2018.5182 PMC7009656 32625816

[7] Sethiya NK (2016). Review on natural growth promoters available for improving gut health of poultry: An alternative to antibiotic growth promoters. Asian J Poultry Sci.

[8] Rafiq K, Tofazzal Hossain M, Ahmed R, Hasan MM, Islam R, Hossen MI (2022). Role of Different Growth Enhancers as Alternative to In-feed Antibiotics in Poultry Industry. Front Vet Sci..

[9] Abbas Hilmi HT, Surakka A, Apajalahti J, Saris PEJ (2007). Identification of the most abundant Lactobacillus species in the crop of 1- and 5-week-old broiler chickens. Appl Environ Microbiol.

[10] Wang L, Fang M, Hu Y, Yang Y, Yang M, Chen Y (2014). Characterization of the most abundant Lactobacillus species in chicken gastrointestinal tract and potential use as probiotics for genetic engineering. Acta Biochim Biophys Sin (Shanghai).

[11] Walter J (2008). Ecological role of lactobacilli in the gastrointestinal tract: Implications for fundamental and biomedical research. Appl Environ Microbiol.

[12] Duar RM, Lin XB, Zheng J, Martino ME, Grenier T, Perez-Munoz M (2017). Lifestyles in transition: Evolution and natural history of the genus Lactobacillus. FEMS Microbiol Rev..

[13] Frese SA, Benson AK, Tannock GW, Loach DM, Kim J, Zhang M, et al. The evolution of host specialization in the vertebrate gut symbiont *Lactobacillus reuteri*. PLoS Genet. 2011;7:e1001314. 10.1371/journal.pgen.1001314 PMC3040671 21379339

[14] Nakphaichit M, Thanomwongwattana S, Phraephaisarn C, Sakamoto N, Keawsompong S, Nakayama J (2011). The effect of including Lactobacillus reuteri KUB-AC5 during post-hatch feeding on the growth and ileum microbiota of broiler chickens. Poult Sci.

[15] Nakphaichit M, Sobanbua S, Siemuang S, Vongsangnak W, Nakayama J, Nitisinprasert S (2019). Protective effect of lactobacillus reuteri KUB-AC5 against salmonella enteritidis challenge in chickens. Benef Microbes.

[16] Schneitz C (2005). Competitive exclusion in poultry - 30 years of research. Food Control..

[17] Hou C, Zeng X, Yang F, Liu H, Qiao S (2015). Study and use of the probiotic Lactobacillus reuteri in pigs: A review. J Anim Sci Biotechnol.

[18] Cleusix V, Lacroix C, Vollenweider S, Duboux M, Le Blay G (2007). Inhibitory activity spectrum of reuterin produced by Lactobacillus reuteri against intestinal bacteria. BMC Microbiol.

[19] Asare PT, Greppi A, Stettler M, Schwab C, Stevens MJA, Lacroix C (2018). Decontamination of Minimally-Processed Fresh Lettuce Using Reuterin Produced by Lactobacillus reuteri. Front Microbiol..

[20] Asare PT, Zurfluh K, Greppi A, Lynch D, Schwab C, Stephan R (2020). Reuterin Demonstrates Potent Antimicrobial Activity Against a Broad Panel of Human and Poultry Meat Campylobacter spp. Isolates Microorganisms.

[21] Engels C, Ruscheweyh HJ, Beerenwinkel N, Lacroix C, Schwab C (2016). The common gut microbe Eubacterium hallii also contributes to intestinal propionate formation. Front Microbiol..

[22] Ramirez Garcia A, Zhang J, Greppi A, Constancias F, Wortmann E, Wandres M (2021). Impact of manipulation of glycerol/diol dehydratase activity on intestinal microbiota ecology and metabolism. Environ Microbiol.

[23] Walter J, Britton RA, Roos S (2011). Host-microbial symbiosis in the vertebrate gastrointestinal tract and the Lactobacillus reuteri paradigm. Proc Natl Acad Sci U S A..

[24] Greppi A, Asare PT, Schwab C, Zemp N, Stephan R, Lacroix C (2020). Isolation and Comparative Genomic Analysis of Reuterin-Producing Lactobacillus reuteri From the Chicken Gastrointestinal Tract. Front Microbiol.

[25] Vollenweider S, Evers S, Zurbriggen K, Lacroix C (2010). Unraveling the hydroxypropionaldehyde (HPA) system: An active antimicrobial agent against human pathogens. J Agric Food Chem.

[26] Dozier IWA, Kerr BJ, Branton SL (2011). Apparent metabolizable energy of crude glycerin originating from different sources in broiler chickens. Poult Sci.

[27] Groesbeck CN, McKinney LJ, DeRouchey JM, Tokach MD, Goodband RD, Dritz SS (2008). Effect of crude glycerol on pellet mill production and nursery pig growth performance. J Anim Sci.

[28] Topal E, Ozdogan M (2013). Effects of glycerol on the growth performance, internal organ weights, and drumstick muscle of broilers. J Appl Poultry Res.

[29] Wang A, Anderson D, Rathgeber B (2018). Using different levels of glycerine, glucose, or sucrose in broiler starter diets to overcome negative effects of delayed feed access on growth performance. Can J Anim Sci.

[30] Asare PT, Greppi A, Pennacchia A, Brenig K, Geirnaert A, Schwab C (2021). In vitro Modeling of Chicken Cecal Microbiota Ecology and Metabolism Using the PolyFermS Platform. Front Microbiol.

[31] Engels C, Schwab C, Zhang J, Stevens MJA, Bieri C, Ebert M-O (2016). Acrolein contributes strongly to antimicrobial and heterocyclic amine transformation activities of reuterin. in revision. Nat Publishing Group..

[32] Casas IA, Dobrogosz WJ (2000). Validation of the Probiotic Concept: Lactobacillus reuteri confers broad-spectrum protection against disease in humans and animals. Microb Ecol Health Dis..

[33] Cleusix V, Lacroix C, Vollenweider S, Le Blay G (2008). Glycerol induces reuterin production and decreases Escherichia coli population in an in vitro model of colonic fermentation with immobilized human feces. FEMS Microbiol Ecol.

[34] Yang X, Yin F, Yang Y, Lepp D, Yu H, Ruan Z (2018). Dietary butyrate glycerides modulate intestinal microbiota composition and serum metabolites in broilers. Sci Rep.

[35] Dishisha T, Pereyra LP, Pyo S-H, Britton RA, Hatti-Kaul R (2014). Flux analysis of the Lactobacillus reuteri propanediol-utilization pathway for production of 3-hydroxypropionaldehyde, 3-hydroxypropionic acid and 1,3-propanediol from glycerol. Microb Cell Fact.

[36] Biebl H, Menzel K, Zeng AP, Deckwer WD (1999). Microbial production of 1,3-propanediol. Appl Microbiol Biotechnol.

[37] Oakley BB, Lillehoj HS, Kogut MH, Kim WK, Maurer JJ, Pedroso A (2014). The chicken gastrointestinal microbiome. FEMS Microbiol Lett.

[38] Stanley D, Hughes RJ, Geier MS, Moore RJ (2016). Bacteria within the gastrointestinal tract microbiota correlated with improved growth and feed conversion: Challenges presented for the identification of performance enhancing probiotic bacteria. Front Microbiol..

[39] Zhang J, Lacroix C, Wortmann E, Ruscheweyh HJ, Sunagawa S, Sturla SJ (2019). Gut microbial beta-glucuronidase and glycerol/diol dehydratase activity contribute to dietary heterocyclic amine biotransformation. BMC Microbiol.

[40] Hu Z, Guo Y (2007). Effects of dietary sodium butyrate supplementation on the intestinal morphological structure, absorptive function and gut flora in chickens. Anim Feed Sci Technol.

[41] Jerzsele A, Szeker K, Csizinszky R, Gere E, Jakab C, Mallo JJ (2012). Efficacy of protected sodium butyrate, a protected blend of essential oils, their combination, and Bacillus amyloliquefaciens spore suspension against artificially induced necrotic enteritis in broilers. Poult Sci.

[42] Leeson S, Namkung H, Antongiovanni M, Lee EH. Effect of butyric acid on the performance and carcass yield of broiler chickens. Poult Sci. 2005;84:1418–22. 10.1093/ps/84.9.1418 16206563

[43] Fernández-Rubio C, Ordóñez C, Abad-González J, Garcia-Gallego A, Honrubia MP, Mallo JJ (2009). Butyric acid-based feed additives help protect broiler chickens from Salmonella enteritidis infection. Poult Sci.

[44] Zhou ZY, Packialakshmi B, Makkar SK, Dridi S, Rath NC (2014). Effect of butyrate on immune response of a chicken macrophage cell line. Vet Immunol Immunopathol.

[45] Bedford A, Gong J (2018). Implications of butyrate and its derivatives for gut health and animal production. Animal Nutrition.

[46] Yu B, Liu JR, Chiou MY, Hsu YR, Chiou PWS (2007). The effects of probiotic Lactobacillus reuteri Pg4 strain on intestinal characteristics and performance in broilers. Asian-Australas J Anim Sci.

[47] Fuller R, Brooker BE (1974). Lactobacilli which attach to the crop epithelium of the fowl. Am J Clin Nutr.

[48] Guan LL, Hagen KE, Tannock GW, Korver DR, Fasenko GM, Allison GE (2003). Detection and identification of Lactobacillus species in crops of broilers of different ages by using PCR-denaturing gradient gel electrophoresis and amplified ribosomal DNA restriction analysis. Appl Environ Microbiol.

[49] Michel C, Kravtchenko TP, David A, Gueneau S, Kozlowski F, Cherbut C (1998). In Vitro prebiotic effects of Acacia gums onto the human intestinal microbiota depends on both botanical origin and environmental pH. Anaerobe.

[50] Bolyen E, Rideout JR, Dillon MR, Bokulich NA, Abnet CC, Al-Ghalith GA (2019). Reproducible, interactive, scalable and extensible microbiome data science using QIIME 2. Nat Biotechnol.

[51] Callahan BJ, McMurdie PJ, Rosen MJ, Han AW, Johnson AJA, Holmes SP (2016). DADA2: High-resolution sample inference from Illumina amplicon data. Nat Methods.

[52] Rognes T, Flouri T, Nichols B, Quince C, Mahé F (2016). VSEARCH: A versatile open source tool for metagenomics. PeerJ.

[53] Price MN, Dehal PS, Arkin AP. FastTree 2 - Approximately maximum-likelihood trees for large alignments. PLoS One. 2010;5:e9490. 10.1371/journal.pone.0009490 PMC2835736 20224823

[54] Katoh K, Standley DM (2013). MAFFT multiple sequence alignment software version 7: Improvements in performance and usability. Mol Biol Evol.

[55] Quast C, Pruesse E, Yilmaz P, Gerken J, Schweer T, Yarza P (2013). The SILVA ribosomal RNA gene database project: Improved data processing and web-based tools. Nucleic Acids Res.

[56] Bokulich NA, Kaehler BD, Rideout JR, Dillon M, Bolyen E, Knight R (2018). Optimizing taxonomic classification of marker-gene amplicon sequences with QIIME 2’s q2-feature-classifier plugin. Microbiome.

[57] Robeson Michael S (2020). DRO. RESCRIPt: Reproducible sequence taxonomy reference database management for the masses. SELL J..

[58] Love MI, Huber W, Anders S (2014). Moderated estimation of fold change and dispersion for RNA-seq data with DESeq2. Genome Biol.

