# Supplementary material for: Glycerol and reuterin-producing Limosilactobacillus reuteri enhance butyrate production and inhibit Enterobacteriaceae in broiler chicken cecal microbiota PolyFermS model
Source: BMC Microbiol. 2023 Dec 5;23:384. doi: 10.1186/s12866-023-03091-6 (PMC10696668; doi:10.1186/s12866-023-03091-6)
Supplement: Supplementary file 1 — Additional file 1: Table S1. Primers used for the detection of bacterial groups by qPCR and for the PCR amplification step of 16S rRNA amplicon Illumina sequencing. Figure S1. Daily metabolite concentrations in the effluent of control and treatment reactors during F1 measured by HPLC-IR: control reactor) (A), 50 mM glycerol (50G) supplementation (B), and 100 mM glycerol (100G) supplementation (C). Lactate was below detection limit. Figure S2. Daily metabolite concentrations in the effluent of control and treatment reactors during F2 measured by HPLC-IR: control reactor) (A), L. reuteri (Lbr) supplementation (B), 100 mM glycerol (100G) supplementation (C), and L. reuteri and 100 mM glycerol (Lbr-100G) supplementation (D). Lactate was below detection limit. Figure S3. Daily metabolite concentrations in the effluent of control and treatment reactors during F3 measured by HPLC-IR: control reactor) (A), L. reuteri (Lbr) supplementation (B), 100 mM glycerol (100G) supplementation (C), and L. reuteri and 100 mM glycerol (Lbr-100G) supplementation (D). Lactate was below detection limit. Figure S4. Daily quantification of key bacterial populations in the effluent of control and treatment reactors during F1 measured by qPCR: control reactor) (A), 50 mM glycerol (50G) supplementation (B), and 100 mM glycerol (100G) supplementation (C). Figure S5. Daily quantification of key bacterial populations in the effluent of control and treatment reactors during F2 measured by qPCR: control reactor) (A), L. reuteri (Lbr) supplementation (B) 100 mM glycerol (100G) supplementation (C), and L. reuteri and 100 mM glycerol (Lbr-100G) supplementation (D). Figure S6. Daily quantification of key bacterial populations in the effluent of control and treatment reactors during F3 measured by qPCR: control reactor) (A), L. reuteri (Lbr) supplementation (B) 100 mM glycerol (100G) supplementation (C), and L. reuteri and 100 mM glycerol (Lbr-100G) supplementation (D). Figure S7. Genus taxa with differen [file 12866_2023_3091_MOESM1_ESM.docx]

**SUPPLEMENTARY MATERIAL**

**Glycerol and reuterin-producing *Limosilactobacillus reuteri* enhance butyrate production and inhibit *Enterobacteriaceae* in broiler chicken cecal microbiota PolyFermS model**

Paul Tetteh Asare^1,2+^, Anna Greppi^1+^, Annelies Geirnaert^1^, Alessia Pennacchia^1^, Angela Babst^1^, Christophe Lacroix^1*^

^1^ Laboratory of Food Biotechnology, Institute of Food, Nutrition and Health, Department of Health Sciences and Technology, ETH Zürich, Zürich, Switzerland

^2^ Present address: Gnubiotics Sciences SA, Epalinges, Switzerland

- These authors contributed equally

* Corresponding author: Christophe Lacroix, Laboratory of Food Biotechnology, LFV D 20, Schmelzbergstrasse 7, CH-8042 Zurich

christophe.lacroix@hest.ethz.ch

**Table S1**: Primers used for the detection of bacterial groups by qPCR and for the PCR amplification step of 16S rRNA amplicon Illumina sequencing.

| **Primer** | **Sequence 5’ – 3’** | **Target** | **Reference** |
| --- | --- | --- | --- |
| Eub338F  Eub518R | ACT CCT ACG GGA GGC AGC AG  ATT ACC GCG GCTVGCT GG | Total bacteria | [1] |
| Firm 934F  Firm 1060R | GGA GYA TGT GGT TTA ATT CGA AGC A  AGC TGA CGA CAA CCA TGC AC | Firmicutes | [2] |
| Bac303F  Bfr-Femrev | GAA GGT CCC CCA CAT TG  CGC KAC TTG GCT GGT TCA G | Bacteroidetes | [2] |
| RumiF  RumiR | ACTGAGAGGTTGAACGGCCA  CCTTTACACCCAGTAAWTCCGGA | *Ruminococcaceae* | [3] |
| F_Lacto 05  R_Lacto 04 | AGC AGT AGG GAA TCT TCC A  CGC CAC TGG TGT TCY TCC ATA TA | *Lactobacillus-Leuconostoc-Pediococcus* spp. | [4] |
| Bifi_F  Bifi_R | TCG CGT CYG GTG TGA AAG  CCA CAT CCA GCR TCC AC | *Bidfidobacteriaceae* | [5] |
| Eco1457F  Eco1652R | CAT TGA CGT TAC CCG CAG AAG AAG CCTC TAC GAG ACT CAA GCT TGC | *Enterobacteriaceae* | [6] |
| pduC_F  pduC_R | CCTGAAGTAAAYCGCATCTT  GAAACYATTTCAGTTTATGG | Reuterin-producing *L. reuteri* | [7] |
| 338F  518R | ACWCCTACGGGWGGCAGCAG  ATTACCGCGGCTGCTGG | V3 region, 16Sr RNA gene | [8] |
| 515F  806R | GTGCCAGCMGCCGCGGTAA  GGACTACHVGGGTWTCTAAT | V4 region, 16S rRNA gene | [9] |


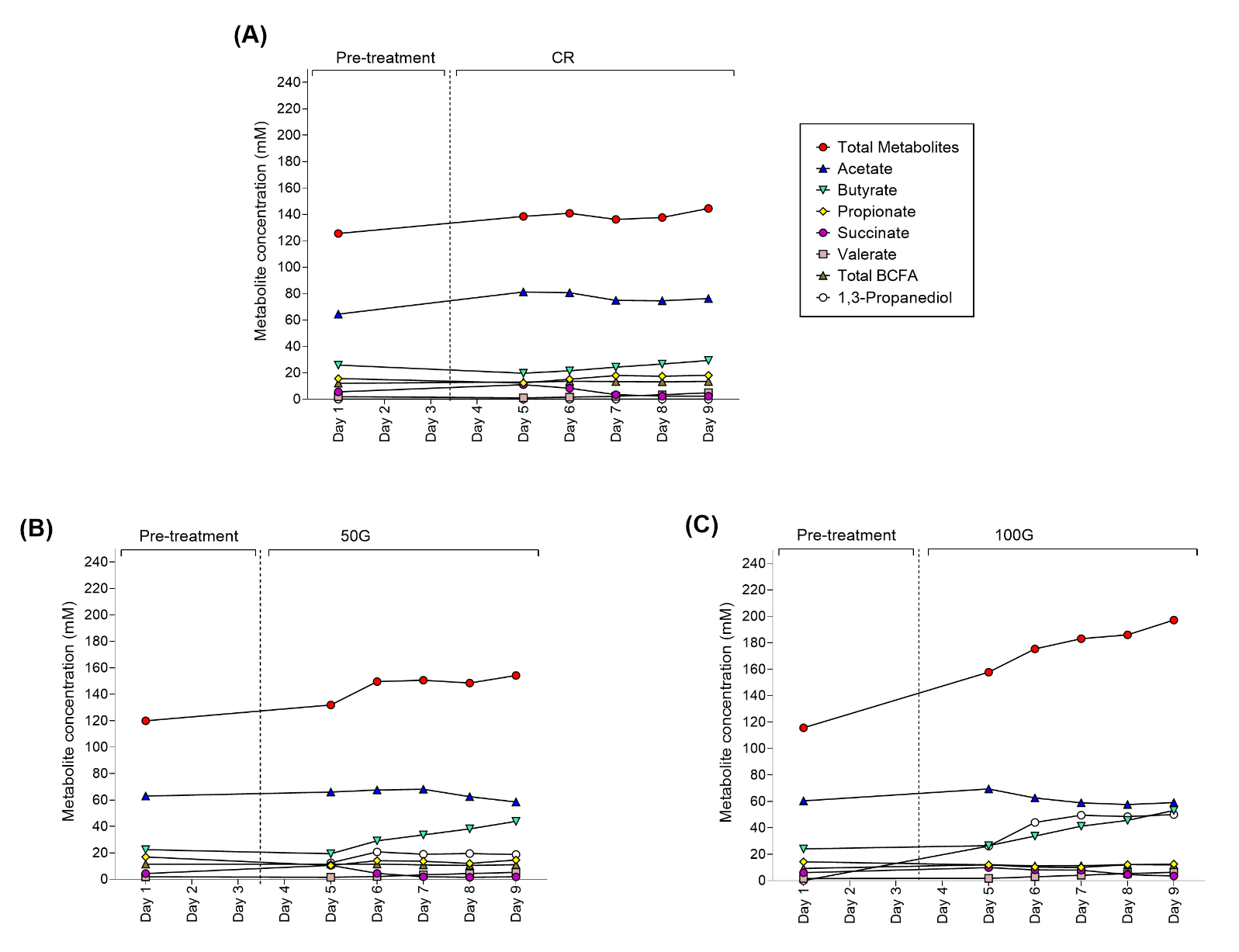


**Figure S1.** Daily metabolite concentrations in the effluent of control and treatment reactors during F1 measured by HPLC-IR: control reactor) (**A**), 50 mM glycerol (50G) supplementation (**B**), and 100 mM glycerol (100G) supplementation (**C**). Lactate was below detection limit.

**
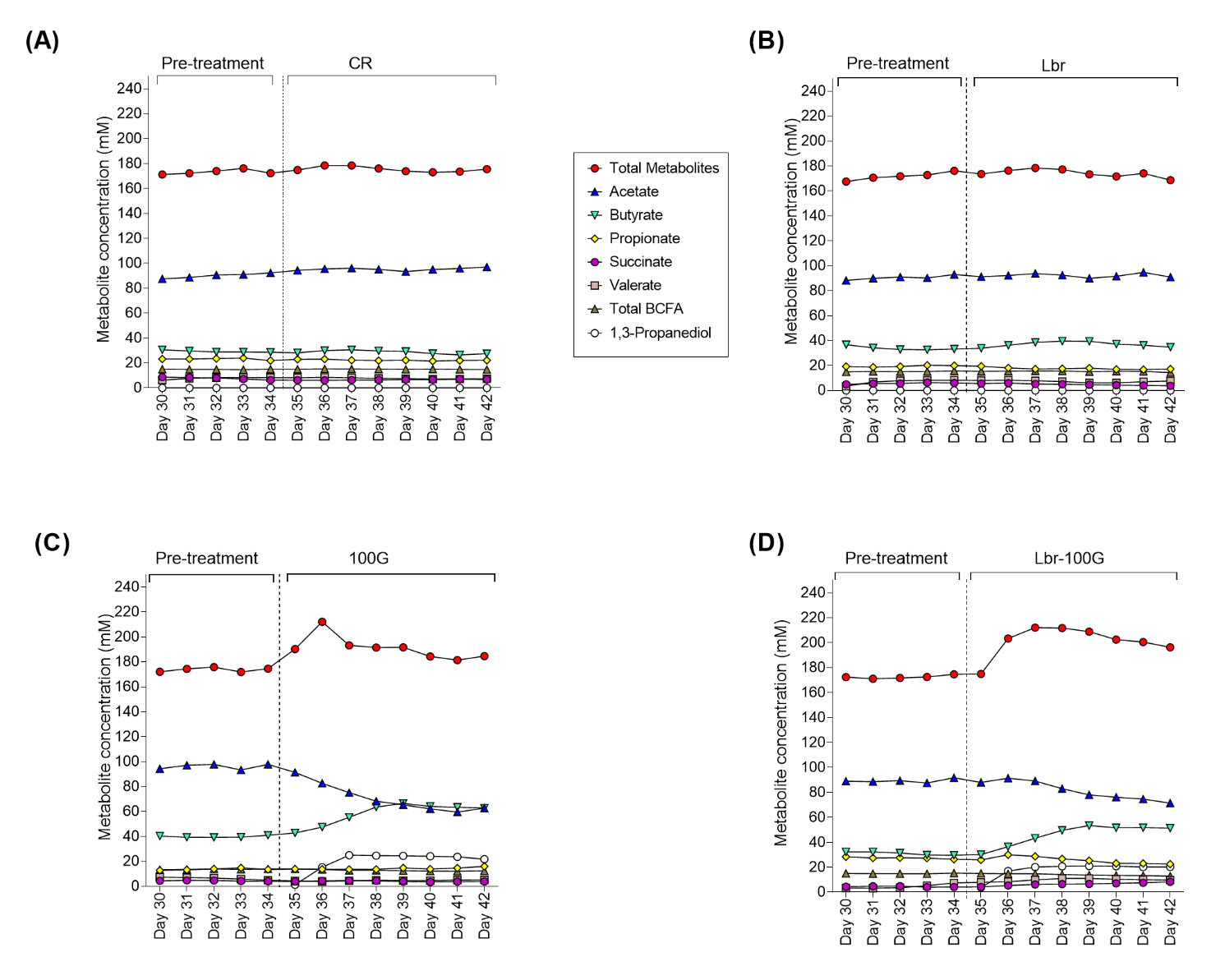
**

**Figure S2.** Daily metabolite concentrations in the effluent of control and treatment reactors during F2 measured by HPLC-IR: control reactor) (**A**), *L. reuteri* (Lbr) supplementation (**B**), 100 mM glycerol (100G) supplementation (**C**), and *L. reuteri* and 100 mM glycerol (Lbr-100G) supplementation (**D**). Lactate was below detection limit.

**
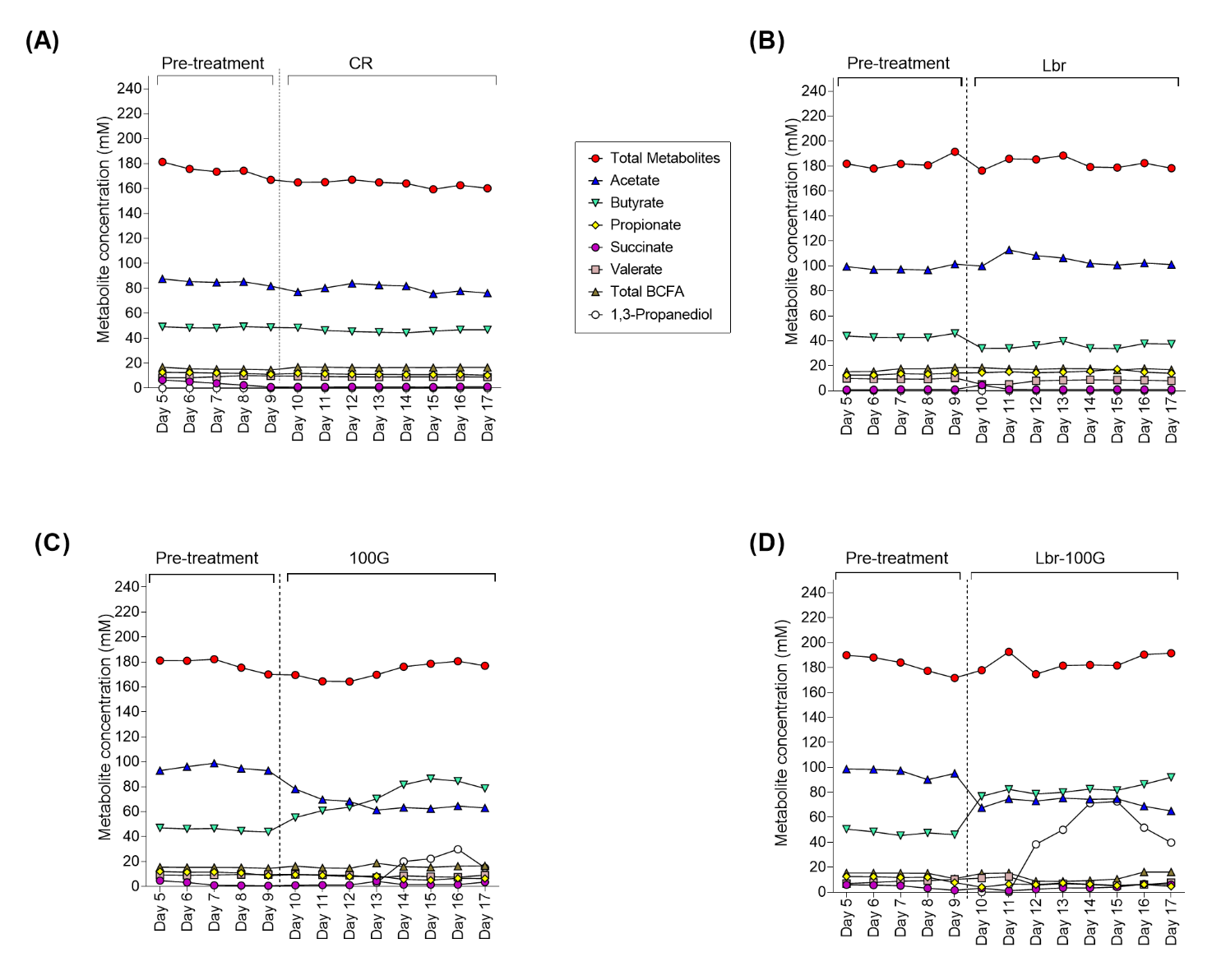
**

**Figure S3.** Daily metabolite concentrations in the effluent of control and treatment reactors during F3 measured by HPLC-IR: control reactor) (**A**), *L. reuteri* (Lbr) supplementation (**B**), 100 mM glycerol (100G) supplementation (**C**), and *L. reuteri* and 100 mM glycerol (Lbr-100G) supplementation (**D**). Lactate was below detection limit.

**
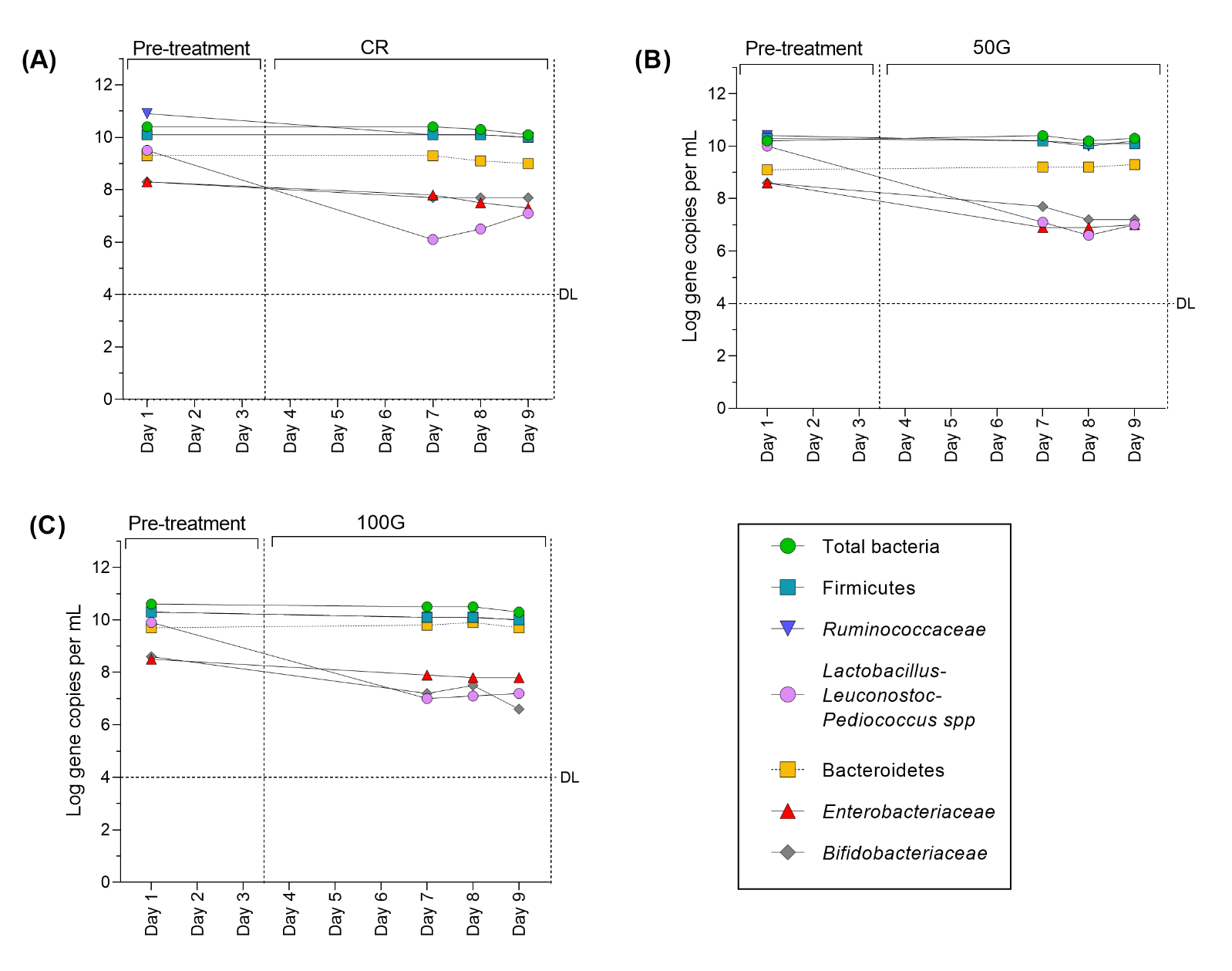
**

**Figure S4.** Daily quantification of key bacterial populations in the effluent of control and treatment reactors during F1 measured by qPCR: control reactor) (**A**), 50 mM glycerol (50G) supplementation (**B**), and 100 mM glycerol (100G) supplementation (**C**).

**
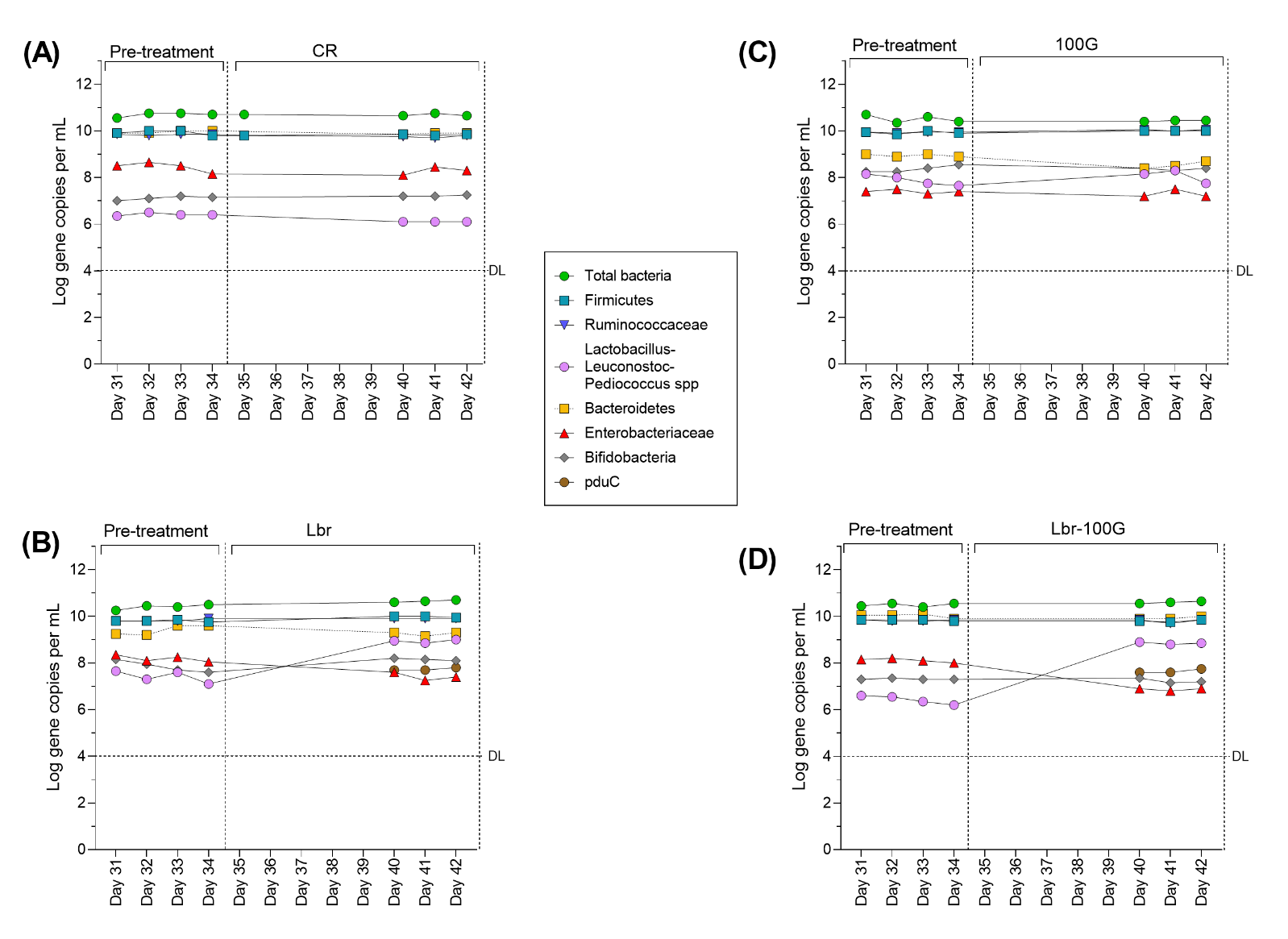
**

**Figure S5.** Daily quantification of key bacterial populations in the effluent of control and treatment reactors during F2 measured by qPCR: control reactor) (**A**), *L. reuteri (*Lbr) supplementation (**B**) 100 mM glycerol (100G) supplementation (**C**), and *L. reuteri* and 100 mM glycerol (Lbr-100G) supplementation (**D**).

**
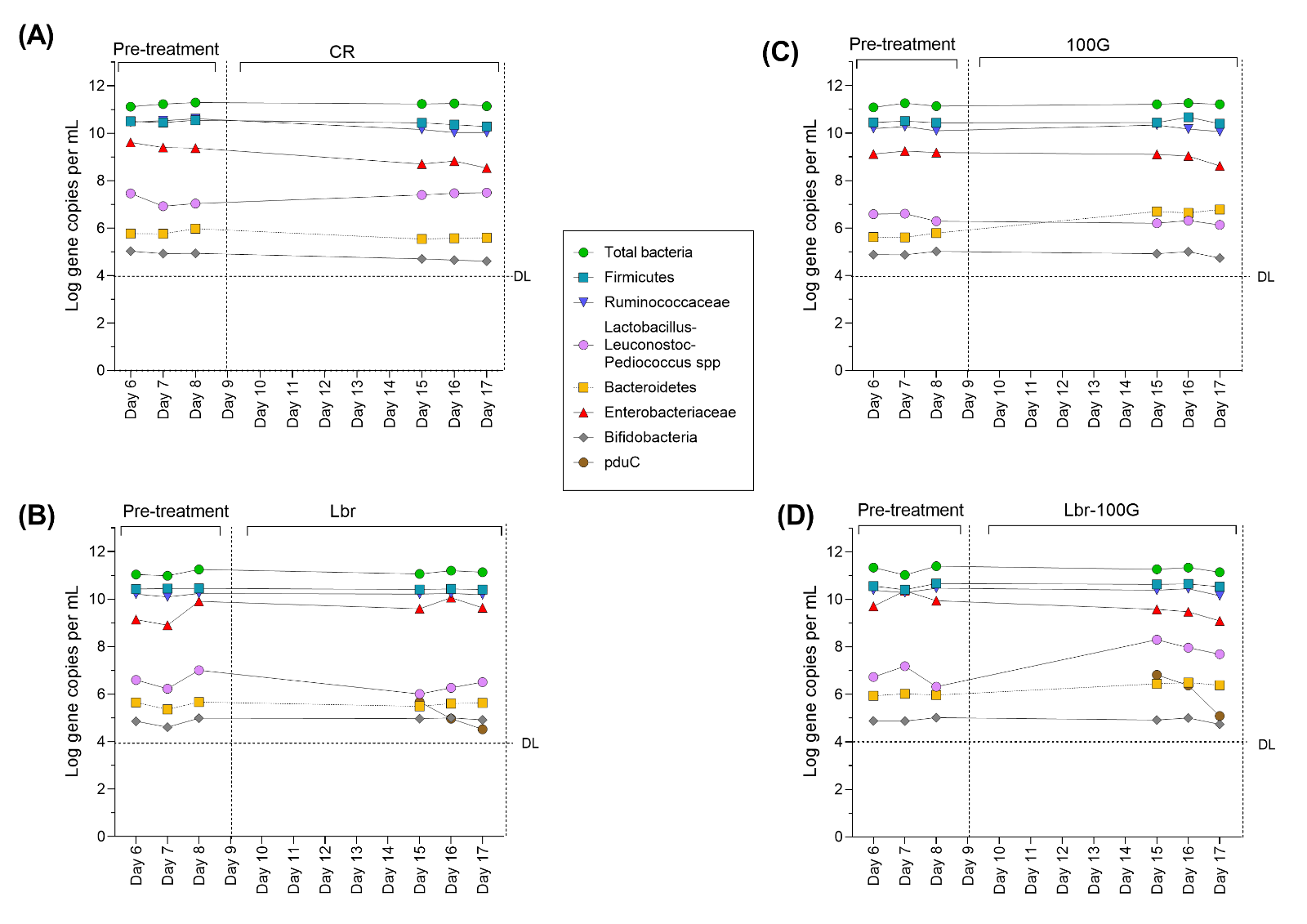
**

**Figure S6.** Daily quantification of key bacterial populations in the effluent of control and treatment reactors during F3 measured by qPCR: control reactor) (**A**), *L. reuteri (*Lbr) supplementation (**B**) 100 mM glycerol (100G) supplementation (**C**), and *L. reuteri* and 100 mM glycerol (Lbr-100G) supplementation (**D**).

**
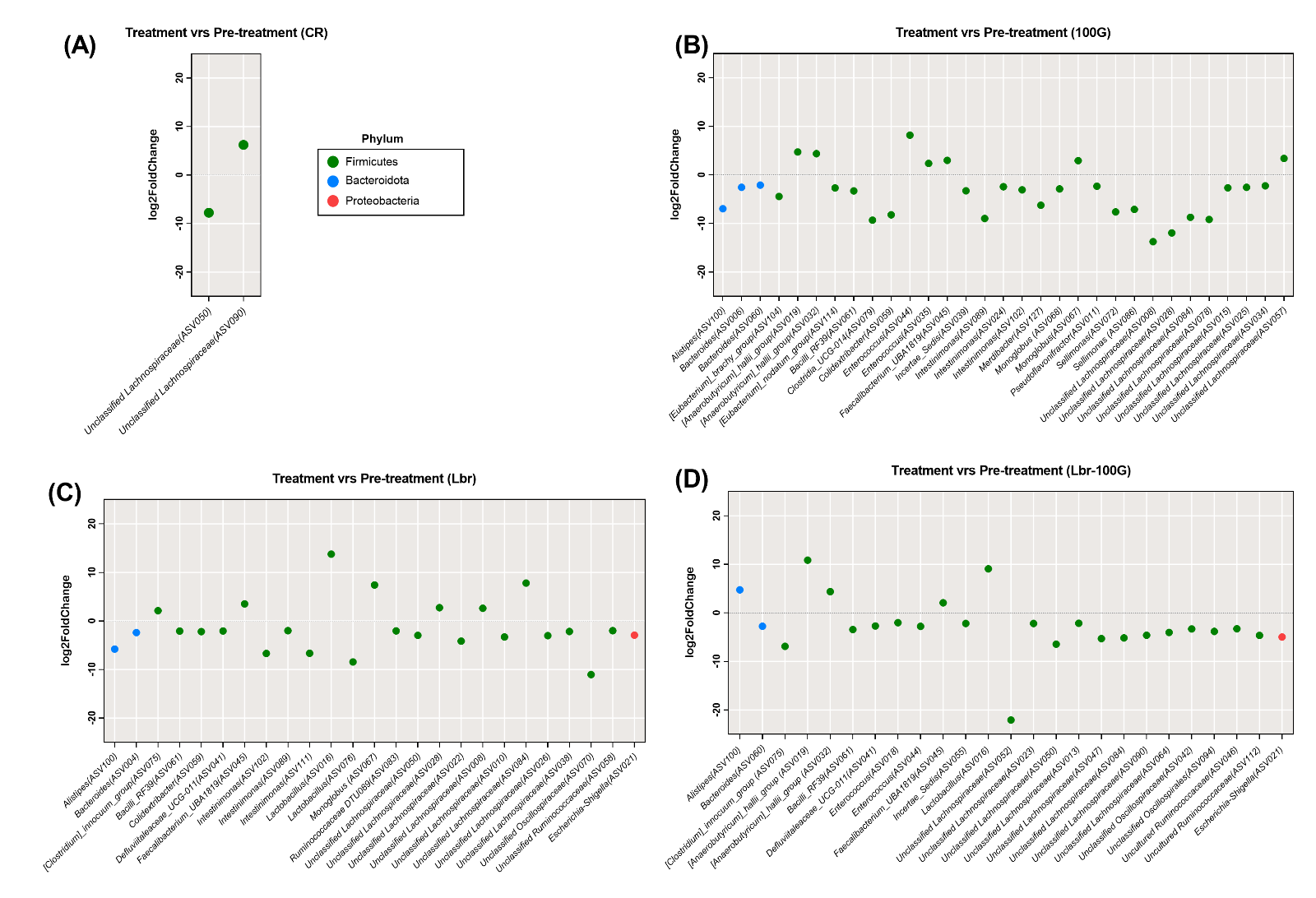
**

**Figure S7.** Genus taxa with differential relative abundance in reactor effluent during treatment compared to pre-treatment (DeSeq2 analysis) in modelled cecal microbiota F2. Taxa are ordered according to log2 fold-change. Each ASV affected for a given genus is indicated with a dot. Dots are coloured according to phylum with the color code in the graph legend. Only taxa with more than 2 log2 fold-change and which are significantly differentially abundant (LRT, *P* ≤ 0.05) are shown. CR, Control reactor; 100G, 100 mM glycerol ; Lbr, *L. reuteri* PTA5_F13; Lbr-100G*, L. reuteri* PTA5_F13 and 100 mM glycerol.

**
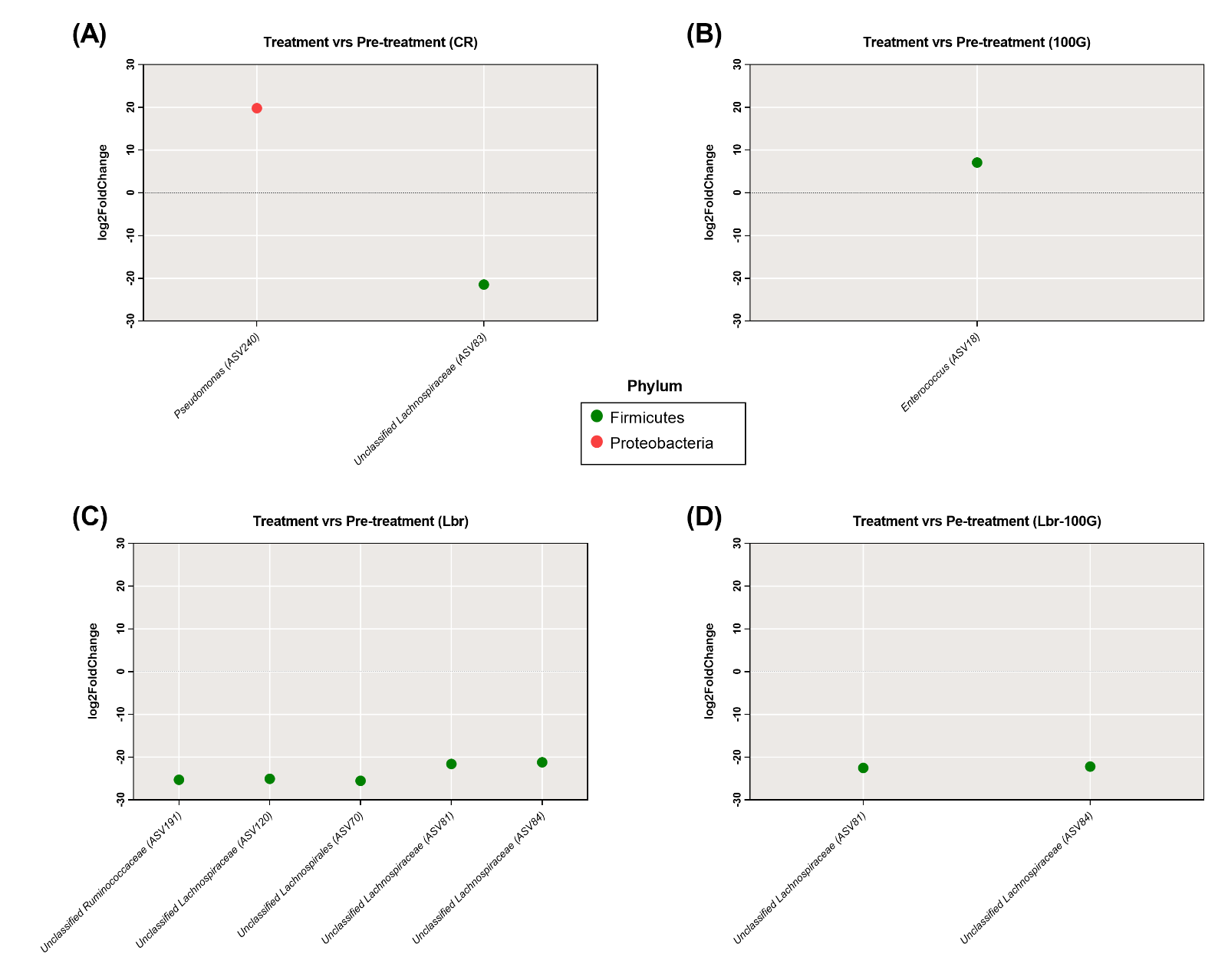
**

**Figure S8**. Genus taxa with differential relative abundance in reactor effluent during pre-treatment compared with treatment (DeSeq2 analysis) in modelled cecal microbiota F3. Taxa are ordered according to log2 fold-change. Each ASV affected for a given genus is indicated with a dot. Dots are coloured according to their phylum with the color code in the graph legend. Only taxa with at least 2 log2 fold-change and which are significantly differentially abundant (LRT, P ≤ 0.05) are shown. CR, Control reactor; 100G, 100 mM glycerol ; Lbr, *L. reuteri* PTA5_F13; Lbr-100G, *L. reuteri* PTA5_F13 and 100 mM glycerol.

**Reference**

1. Guo X, Xia X, Tang R, Zhou J, Zhao H, Wang K. Development of a real-time PCR method for Firmicutes and Bacteroidetes in faeces and its application to quantify intestinal population of obese and lean pigs. Lett Appl Microbiol. 2008;47:367–73.

2. Ramirez-Farias C, Slezak K, Fuller Z, Duncan A, Holtrop G, Louis P. Effect of inulin on the human gut microbiota: stimulation of Bifidobacterium adolescentis and Faecalibacterium prausnitzii. British Journal of Nutrition. 2009;101:533–40.

3. Garcia-Mazcorro JF, Suchodolski JS, Jones KR, Clark-Price SC, Dowd SE, Minamoto Y, et al. Effect of the proton pump inhibitor omeprazole on the gastrointestinal bacterial microbiota of healthy dogs. FEMS Microbiol Ecol. 2012;80:624–36.

4. Furet J-P, Firmesse O, Gourmelon M, Bridonneau C, Tap J, Mondot S, et al. Comparative assessment of human and farm animal faecal microbiota using real-time quantitative PCR. FEMS Microbiol Ecol. 2009;68:351–62.

5. Meimandipour A, Shuhaimi M, Soleimani AF, Azhar K, Hair-Bejo M, Kabeir BM, et al. Selected microbial groups and short-chain fatty acids profile in a simulated chicken cecum supplemented with two strains of Lactobacillus. Poult Sci. 2010;89:470–6.

6. Bartosch S, Fite A, Macfarlane GT, Mcmurdo MET. Characterization of Bacterial Communities in Feces from Healthy Elderly Volunteers and Hospitalized Elderly Patients by Using Real-Time PCR and Effects of Antibiotic Treatment on the Fecal Microbiota Characterization of Bacterial Communities in Feces from. Appl Environ Microbiol. 2004;70:3575–81.

7. Walter J, Britton RA, Roos S. Host-microbial symbiosis in the vertebrate gastrointestinal tract and the Lactobacillus reuteri paradigm. Proc Natl Acad Sci U S A. 2011;108 Suppl Supplement_1:4645–52.

8. Muyzer G, de Waal EC, Uitterlinden AG. Profiling of complex microbial populations by denaturing gradient gel electrophoresis analysis of polymerase chain reaction-amplified genes coding for 16S rRNA. Appl Environ Microbiol. 1993;59:695–700.

9. Caporaso JG, Lauber CL, Walters WA, Berg-Lyons D, Lozupone CA, Turnbaugh PJ, et al. Global patterns of 16S rRNA diversity at a depth of millions of sequences per sample. Proc Natl Acad Sci U S A. 2011;108 SUPPL. 1:4516–22.
